# Nosocomial Transmission of Necrotizing Fasciitis: A Molecular Characterization of Group A Streptococcal DNases in Clinical Virulence

**DOI:** 10.3390/microorganisms12112209

**Published:** 2024-10-31

**Authors:** Geoffrey Deneubourg, Lionel Schiavolin, Dalila Lakhloufi, Gwenaelle Botquin, Valérie Delforge, Mark R. Davies, Pierre R. Smeesters, Anne Botteaux

**Affiliations:** 1 Molecular Bacteriology, European Plotkin Institute for Vaccinology (EPIV), Université Libre de Bruxelles, 1070 Bruxelles, Belgium; geoffrey.deneubourg@ulb.be (G.D.); lionel.schiavolin@ulb.be (L.S.); dalila.lakhloufi@ulb.be (D.L.); gwenaelle.botquin@ulb.be (G.B.); valerie.delforge@ulb.be (V.D.); pierre.smeesters@hubruxelles.be (P.R.S.); 2 Department of Microbiology and Immunology, The University of Melbourne at the Peter Doherty Institute for Infection and Immunity, Melbourne, Victoria 3000, Australia; mark.davies1@unimelb.edu.au; 3 Department of Pediatrics, Academic Children Hospital Queen Fabiola, Brussels University Hospital, Université Libre de Bruxelles, 1020 Bruxelles, Belgium

**Keywords:** host–pathogen interactions, bacterial virulence, *Streptococcus pyogenes*, DNases, necrotizing fasciitis

## Abstract

*Streptococcus pyogenes*, or Group A *Streptococcus* (GAS), is responsible for over 500,000 deaths per year. Approximately 15% of these deaths are caused by necrotizing soft-tissue infections. In 2008, we isolated an M5 GAS, named the LO1 strain, responsible for the nosocomial transmission of necrotizing fasciitis between a baby and a nurse in Belgium. To understand this unusual transmission route, the LO1 strain was sequenced. A comparison of the LO1 genome and transcriptome with the reference M5 Manfredo strain was conducted. We found that the major differences were the presence of an additional DNase and a Tn916-like transposon in the LO1 and other invasive M5 genomes. RNA-seq analysis showed that genes present on the transposon were barely expressed. In contrast, the DNases presented different expression profiles depending on the tested conditions. We generated knock-out mutants in the LO1 background and characterized their virulence phenotype. We also determined their nuclease activity on different substrates. We found that DNases are dispensable for biofilm formation and adhesion to both keratinocytes and pharyngeal cells. Three of these were found to be essential for blood survival; Spd4 and Sdn are implicated in phagocytosis resistance, and Spd1 is responsible for neutrophil extracellular trap (NET) degradation.

## 1. Introduction

*Streptococcus pyogenes*, also known as Group A *Streptococcus* or GAS, is a Gram-positive bacterium and a strictly human pathogen. GAS is responsible for a wide spectrum of diseases ranging from superficial diseases such as pharyngitis and impetigo to invasive diseases such as necrotizing fasciitis (NF) or streptococcal toxic shock syndrome (STSS). GAS can also cause post-infection auto-immune sequelae such as rheumatic heart disease or glomerulonephritis [1,2]. Each year, GAS is responsible for approximately 700 million cases of superficial infection and 500,000 deaths worldwide [3].

GAS possesses an array of chromosomal- and phage-encoded virulence factors that differ from strain to strain in both content and expression regulation [1,4,5]. One of the most studied virulence factors is the M protein, encoded by the *emm* gene, which is one of the leading candidate vaccine antigens. The M protein allows bacteria to evade the immune system and adhere to host cells. Its hypervariable N-terminal region (HVR) is used for GAS typing, known as *emm*-typing [6]. This diversity of sequences leads to the existence of at least 261 *emm*-types impairing the development of broad coverage HVR M protein-based vaccines [7,8].

During GAS evolution, lysogenic phages have played an important role in shaping the virulence of GAS. Indeed, phages allow GAS to gain important genes such as virulence factors (superantigens, DNases, phospholipase, etc.) and antibiotic resistance genes (such as *mefA*) [9]. Among phage-encoded virulence factors, DNases, also known as streptodornases, are key players in GAS virulence. A study of 568 M28 GAS strains isolated from invasive (iGAS) and pharyngitis cases identified 29 distinct phage-encoded virulence genes [10]. Eighty-four percent of these strains contained a prophage carrying the *spd1* and *speC* toxin genes. More recently, a novel lineage of M3 was associated with the majority of iGAS isolates during the 2008–2009 upsurge period in the UK [11]. This strain was also characterized by the presence of *spd1* and *speC*. A new variant of GAS serotype M1 (designated ‘M1_UK_’) has been reported in the UK, linked with seasonal scarlet fever surge, increase in invasive infections, and enhanced expression of the superantigen SpeA [12]. Recently, genomic analysis of the M1_UK_ lineage in Australia showed the existence of sub-lineages containing a novel virulence gene set composed of *ssa*, *speC*, and *spd1* [13]. To date, eight DNases have been identified in GAS [14]. SpnA and SpdB are chromosomally encoded, whereas Sda1, Sda2, Spd1, Spd3, Spd4, and Sdn, are prophage encoded. Only SpnA is anchored to the cell wall, whereas others are all secreted [14]. The most studied DNase in GAS virulence is Sda1 [15]. Sda1 allows GAS to avoid neutrophils killing by degrading neutrophil extracellular traps (NETs) [16,17]. This function is also shared by SpnA [18]. Sda1 prevents also TLR9-dependent recognition of GAS inside macrophages by degrading its own genetic material [19]. The only GAS DNases that have been structurally characterized are Spd1 and Sda1 [20,21]. Spd1 belongs to the ββα-metal-dependent nuclease family, with a conserved RGH active site sequence motif. It has been shown that the active site Histidine is crucial for GAS nuclease activity as H121A and H121N mutations abrogate Sda1 activity [20].

In 2008, an 8-month-old child with necrotizing fasciitis underwent surgical debridement in Brussels [22]. The nurse in charge of the surgery cut herself with a contaminated scalpel and developed finger necrosis, despite immediate local disinfection. The bacterium isolated from the nurse’s finger was identified as a M5 GAS strain and named the LO1 strain [22]. Because of this unusual clinical presentation, we aimed to elucidate the molecular determinants of LO1 virulence.

## 2. Materials and Methods (1209)

### 2.1. Bacterial Strains, Human Cells, and Growth Conditions

The LO1 [22] and Manfredo [23] strains have been described previously. Top10 *E. coli* strain (Invitrogen, Waltham, MA, USA) was used for cloning and routinely grown at 37 °C, shaken at 220 rpm in the LB medium (Sigma-Aldrich, Darmstadt, Germany). The GAS strains were plated on either blood agar or Todd-Hewitt agar supplemented with 0.5% yeast extract (THY) (Carl Roth, Karlsruhe, Germany). Bacteria were grown statically at 30 °C (overnight (O/N) growth) or at 37 °C (day growth) in a 5% CO_2_ atmosphere in autoclaved THY medium. The antibiotics used include the following: ampicillin (100 μg·mL^−1^ for *E. coli*) (Carl Roth); kanamycin (50 μg.mL^−1^ for *E. coli* and 300 μg.mL^−1^ for GAS)(MP Biomedicals); spectinomycin (100 μg.mL^−1^ for both) (Merck, Darmstad, Germany). The C-medium was prepared as described in [24]. The commercial pool of serum was purchased from MP Biomedicals (Irvine, CA, USA). Detroit 562 (CCL-138™) (ATCC, Manassas, VA, USA), HFF-1 (SCRC-1041™) (ATCC, USA), and HaCaT (300493) (Cytion, Baden-Wurttemberg, Germany) cell lines were used. Cells were cultured in DMEM (Dulbecco’s modified Eagle’s Medium, Thermo Fisher Scientific, Waltham, MA, USA) supplemented with 10% (HaCat and Detroit) or 15% (HFF-1) *v*/*v* fetal bovine serum (FBS) (Merck) at 37 °C with 5% CO_2_.

### 2.2. Genomic Analysis

The LO1 strain was sequenced using the PacBio platform, as previously described [25]. Open reading frames were predicted using DFAST [26]. Genome annotation was performed using Geneious Prime^®^ 2023.2.1 software using reference genomes. Annotations were manually cured using SWISS-MODEL, NCBI domain search, and GAS literature. They were assigned to different classes based on NMPDR (National Microbial Pathogen Database Resource) annotation [27]. PubMLST database was used to determine the sequence type [28]. Whole-genome alignment of different M5 genes was performed using progressiveMauve software v2.4.0 [29]. Protein alignment of virulence factors and TCS was performed using Geneious alignment tool. Promoters of DNase encoding genes were predicted using BPROM [30]. Circular genome maps were generated using BRIG v0.95 (Blast Ring Image Generator) [31].

### 2.3. Generation of Recombinant DNases and Point Mutagenesis

The genes encoding the DNases *sdn*, *spd1*, *spd3*, and *spd4* were amplified using specific primers. The digested PCR product with *Bam*HI*/Xho*I restriction enzymes was ligated into the *Bam*HI*/Xho*I sites of pET-30a (Merck) to fuse the ORF with a C-terminal hexa-histidine motif. Site-directed mutagenesis was performed using the PrimeSTAR^®^ Mutagenesis Basal Kit (TaKaRa Bio, Shiga, Japan). Synonymous mutations were introduced into the primers to facilitate mutation screening by enzymatic restriction. The mutations were confirmed by sequencing (Eurofins, Hamburg, Germany). All the primers used in this study are listed in Table S3.

### 2.4. KO Mutant Generation

We generated an isogenic mutant of *sdn* in the LO1 strain based on Le Breton et al. [32] and Barnett et al. [33], with minor modifications. Briefly, flanking regions (1 kb) of the *sdn* and *apha3* (kanamycin resistance) genes were amplified by PCR using specific primers (Table S3), subcloned into the pTOPO vector (Invitrogen), and cloned into the thermosensitive pLZts vector using *Eco*RI (NEB, Ipswich, MA, USA). pLZts-*sdn*KO was transformed within GAS LO1. Briefly, O/N cultures of the LO1 strain grown in THY supplemented with 20 mM glycine (THY-G) were diluted 1:20 in 50 mL THY-G and grown to the exponential phase (OD_600_ of 0.3–0.4). The bacterial culture was cooled for 30 min on ice and centrifuged at 8500× *g* for 10 min at 4 °C. The pellet was washed three times with 10 mL ice-cold glycerol 10%. The pellet was resuspended in 1 mL ice-cold glycerol. An aliquot of 200 µL of competent cells was transformed with 500 ng of purified plasmid by electroporation. After recovery in THY at 28 °C and 5% CO_2_ for 3 h, serial dilutions were plated on THY agar containing spectinomycin and incubated at 28 °C for 36–48 h. Allelic exchange was performed as previously described [33]. The Δ*spd1*, Δs*pd3*, and Δ*spd4* mutants were generated by double allelic exchange using pKOSpy [34].

### 2.5. DNase Activity

Recombinant DNases were expressed in Top10 and purified by affinity chromatography using HisPur™ Cobalt resin (Thermo Fisher Scientific) according to standard procedures. Each DNase was quantified and diluted to a final concentration of 0.2 mg·mL^−1^. The substrates were prepared as follows: pGEX4T1 (Plasmid DNA) was extracted from the *E. coli* culture using the E.Z.N.A.^®^ Midi Kit (Omega Bio-Tek, Norcross, GA, USA). Human genomic DNA was extracted using the Monarch^®^ Genomic DNA Purification Kit (NEB). Total human RNA (blood) and total bacterial RNA (GAS LO1) were extracted using a Monarch^®^ Total RNA Miniprep kit (NEB). DNase activity was assayed as follows: 10 µL of DNase or GAS supernatant was mixed with 30 ng of nucleic acids in reaction buffer containing Tris-HCl 0.1 M, MgCl_2_ 0.2 M, and CaCl_2_ 0.2 M in a 96-well plate. Plates were incubated for 40 min at 37 °C, and 10 µL of the reaction mixture was colored with a loading dye and loaded on an agarose gel. Alternatively, 50 µL of Sytox Orange^®^ was added to the reaction and incubated for 5 min, and the fluorescence was read at 545/580 nm using a Tristar LB941 fluorimeter (Berthold, Bad Wildbad, Germany).

### 2.6. Bacterial Growth

Overnight cultures of GAS were diluted to an OD_600_ of 0.05 in THY 0.5% (unless otherwise stated) and grown to OD_600_ of 0.3–0.4. To determine bacterial growth in different media, O/N cultures of GAS in THY were centrifuged at 5000× *g* for 10 min. The pellet was washed once with DPBS (Dulbecco’s Phosphate-Buffer Saline) (Thermo Fisher Scientific) and resuspended in test medium. The bacteria were then diluted in the corresponding medium to an OD_600_ of 0.05.

### 2.7. Biofilms Formation

Biofilm formation was determined as previously described [35]. Briefly, a 72 h biofilm was formed in sterile 96-well plates by diluting 1:20 overnight cultures of GAS strains in THY 0.5%. Mature biofilms were then fixed with methanol, washed with DPBS, and stained with 0.2% (*w*/*v*) crystal violet. The incorporated crystal violet was resolubilized with 1% SDS solution, and the absorbance was measured at OD_540_.

### 2.8. Adhesion to Human Cell Lines

Two days before infection, cells were plated at a density of 1.5 × 10^5^ cells in a 24-well plate. Overnight cultures of GAS strains were diluted to an OD_600_ of 0.05 and grown to an OD_600_ of 0.3–0.4. Bacteria were washed once with DPBS and added to the cells at a multiplicity of infection (MOI) of 100 in DMEM + 1% FBS (Merck). The 24-well plates were centrifuged at 200× *g* for 5 min before incubation at 37 °C 5% CO_2_. After two hours of infection, the cells were washed three times with DPBS and detached with Trypsin-EDTA 0.25% before lysis with Triton X-100 (Sigma-Aldrich, Darmstadt, Germany) 0.025%. Together with the inoculum, the lysates were diluted and plated for CFU (colony-forming unit) counting, which corresponded to intracellular and adherent bacteria. The percentage of entry/adhesion was calculated by dividing the number of bacteria on the plate by that of the inoculum.

### 2.9. Whole Blood Killing Assay

The whole blood killing assay was performed as described previously [36] with slight modifications. Briefly, an O/N culture of GAS was diluted 1:100 in THY 1% and grown to OD_600_ of 0.08–0.1. The bacterial cultures were diluted to 10^−4^ and plated for CFU counting (T_0_). Then, 10 µL of this dilution was mixed with 50 µL DPBS and 140 µL fresh blood from anonymous donors in a sterile 96-well round-bottom plate. Plates were incubated at 37 °C with gentle shaking for three hours, and bacteria were counted. For the cytochalasin D assay, 10 µM cytochalasin D (Sigma-Aldrich) was added to the reaction mixture.

### 2.10. RNA Extraction and Sequencing

For each tested condition (Supplementary data, Table S3), the bacterial pellet was resuspended in ‘RNA protect’ and lysed using matrix B beads. All bacterial lysates were centrifuged at 16,000× *g* for 2 min, and the supernatant was processed for RNA extraction using the Monarch^®^ RNA Miniprep Kit (NEB). An additional DNase step was performed using a TURBO DNA-free™ Kit (Invitrogen). After RNA sequencing (Supplementary data), RNA-seq analysis was performed with Geneious Prime^®^ 2023.2.1 software (Supplementary data).

### 2.11. Statistical Analysis

All quantitative data were analyzed and graphed using the GraphPad Prism 8.4.3 software. Statistical details of the experiments are provided in the respective figure legends and in each Methods section pertaining to the specific technique applied.

## 3. Results

### 3.1. Genomic Comparison of the LO1 and Manfredo Strains

The genome of the *emm*-type 5.44 LO1 strain was sequenced using PacBio technology and assembled into a single circular chromosome of 1,897,626 bp in length. A genomic comparison between LO1, the historical M5 Manfredo strain isolated from an acute rheumatic fever (ARF) in 1952 in the United States [37] and three recent Scottish M5 (*emm*5.23) invasive isolates (Table 1) [38]. A recent outbreak of invasive disease due to *emm*5.23 GAS, as well as increased mortality associated with this *emm*-type, has been observed in the UK [39,40].

**Figure 1:**
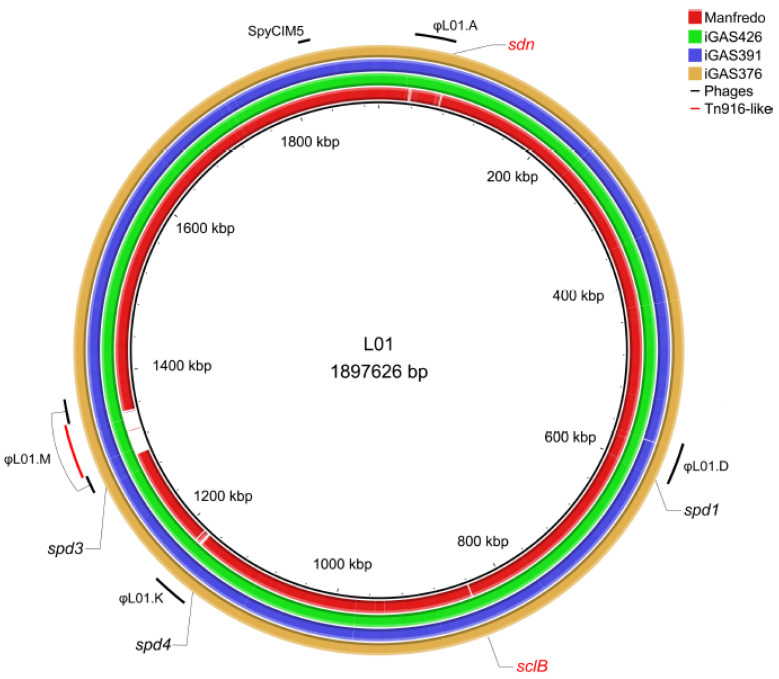
Circular genome map of the Manfredo and 3 Scottish iGAS (iGAS426, iGAS391 and iGAS376) compared to the LO1 genome. Positions of the different prophages (LO1.A, LO1.D, LO1.K, LO1.M and SpyCIM5) of the LO1 are highlighted in black. Position of the Tn916-like transposon is highlighted in red. The φMan.F is not observed as a blank region because several genes are found in the φLO1.A prophage. DNases and *sclB* positions are depicted. The *sdn* (absent) and *sclB* (different in the Manfredo) genes are colored in red. The map was generated using the BRIG software v0.95 [31].

Because prophages play an important role in the evolution and virulence of GAS strains [41], we used PHASTER [42] to detect the presence of prophages in all strains. We found that LO1 and the three Scottish iGAS possess the same four prophages, with one interrupted by a Tn916-like conjugative transposon [43], and one phage satellite (Figure 1). Among the genes present in the Tn916-like transposon, we found *tetR/tetM* genes that could confer tetracycline resistance, a putative hydrolase, and genes that could produce a conjugative pili (type IV secretion system) for its own transfer. These four phages accounted for approximately 14% of the CDSs (266 CDSs). The Manfredo historical strain also contained four prophages and a phage satellite [23], accounting for approximately 14% of its CDSs (267 CDSs). Three prophages were identical between all strains and were annotated based on their chromosomal integration sites [44]: ϕLO1.D and ϕMan.D, which encode DNase Spd1 and the superantigen SpeC; ϕLO1/Man.K, which encodes DNase Spd4; and ϕLO1/Man.M, encoding DNase Spd3, which was disrupted by a transposon in the LO1 strain. While Manfredo contains ϕMan.F, encoding the superantigens SpeH and SpeI, and LO1 contains another prophage, ϕLO1.A, which encodes DNase Sdn (Figure 1). The phage satellite SpyCIM5, present at position S in both strains, did not encode virulence factors [45,46].

**Figure 2:**
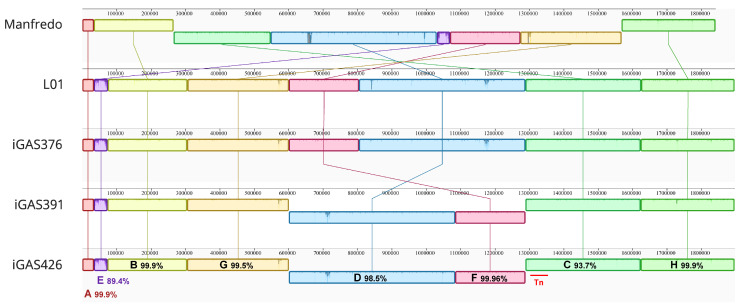
ProgressiveMauve alignment [29] of the Manfredo, LO1 and 3 Scottish iGAS genomes using Geneious Prime^®^ 2023.2.1. The alignment identifies 8 locally collinear blocks (LCBs) annotated from A to H and highlights the different genomic inversions between strains. Pairwise identity of the different LCBs and position of the transposon (red bar) are depicted.

We then aligned the whole genomes of the five strains using progressiveMauve software 2.4.0 [29]. The alignment resulted in eight Locally Collinear Blocks (LCBs) shared among all genomes (Figure 2). The large central inversion observed in the Manfredo genome was reversed in the LO1 and Scottish iGAS (C-D-F-G blocks) (Holden, 2007, Complete genome of acute rheumatic fever-associated serotype M5 *Streptococcus pyogenes* strain Manfredo). A smaller inversion (blocks F and D) was observed in the iGAS391/iGAS426 compared to the Manfredo/LO1/iGAS376 strains. The inversion occurred between iGAS391/iGAS426 phages corresponding to ϕLO1.D and ϕLO1.M which share a 5 kb region containing the tail, hyaluronidase and antireceptor genes. The E box corresponds to the phage encoding *sdn* (ϕLO1.A) in the four iGAS and *speH speI* (ϕMan.F) in Manfredo. Both phages share homology but differ in their regulatory (lysogeny and replication), distal structure (tail, hyaluronidase and antireceptor), lysis, and virulence modules.

As mutations in two-component systems (TCSs), such as CovR/S [47], could result in more invasive phenotypes [48,49], we first retrieved the TCS present in the five M5 genomes based on the list of known GAS TCS [50]. Sequence alignment highlighted differences for two of the 12 identified TCS, FasBCA and YehUT (Supplementary Figure S1). FasBCA TCS, consisting of FasC and FasB which serve as histidine kinases, and FasA, as a response regulator, regulates several virulence factors [51]. The *fasC* gene has a frameshift in the poly(T) stretch, leading to a premature stop codon (at position 172) in the FasC protein of the Manfredo strain. The YehUT TCS controls the expression of the mannose/fructose PTS system [51]. The YehU protein has a leucine at position 133 in Manfredo in place of a phenylalanine in the other strains (Supplementary Figure S1).

In parallel, we analyzed the sequences of virulence factors as SNPs could have an impact on both disease severity and strain tropism [52]. The comparison of known GAS virulence factors content in all five genomes showed that only *speH* and *speI* (from ϕMan.F in Manfredo) and *sdn* (from ϕLO1.A in LO1), already highlighted by the PHASTER analysis, differ between Manfredo and the iGAS. All the virulence factors found in both strains are highly conserved except for the M protein (*emm*5.23 for iGAS, 5.44 for the LO1 and 5.0 for the Manfredo strains) and streptococcal collagen-like surface protein B (SclB). Streptokinase Ska is also truncated due to a premature stop codon in Manfredo, while SclA is truncated in the LO1 and Scottish iGAS strains (Table 2).

Overall, the main differences among the LO1, Scottish iGAS, and Manfredo strains reside in their DNase content and the Tn916 presence. Indeed, LO1 possesses four prophage-encoded DNases, a combination only observed in less than 0.23% of the 2992 available GAS genomes in NCBI (Table S1), in which rare Sdn (10% of the sequenced strains) and Spd4 (6% of the sequenced strains) DNases are present (Supplementary Figure S2). We hypothesized that this specific pattern and/or the presence of Sdn could partially explain the virulence of LO1, which prompted us to study their role.

### 3.2. The spd1, spd3, spd4, and sdn Genes are Differentially Expressed in the LO1 Strain

Before exploring the role of DNases in the LO1 background, we monitored how the four genes were expressed under different clinically relevant conditions. We performed RNA-seq analysis of LO1 and Manfredo strains in laboratory media (Todd-Hewitt Yeast (THY) medium, C-medium, cell culture medium (DMEM)), different phases of growth (exponential, stationary or biofilm), and in the presence of serum or cell lines (HaCaT, HFF-1, and Detroit 562) (Table S2).

**Figure 3:**
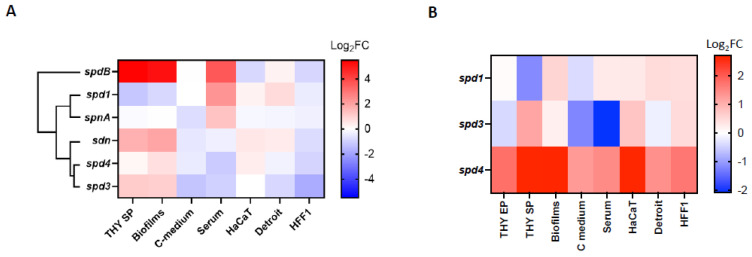
Heatmap of the differential expression (Log_2_FC) of (**A**) the different chromosomally and phage-encoded DNases of the LO1 strain in different culture conditions. Gene expressions in stationary phase (THY SP), biofilms, C-medium, and serum conditions were compared to bacteria grown in exponential phase (THY EP), while HaCat, Detroit, and HFF-1 conditions were compared to bacteria incubated in DMEM + FBS. Hierarchical clustering of DNases according to their expression profiles is displayed. (**B**) The phage-encoded DNases of the LO1 strain compared to Manfredo strain in the same conditions.

We observed that, in the LO1 strain, each DNase gene had a specific profile of expression, with *spd1* having a different expression pattern than the other phage-encoded DNases (Figure 3A). Overall, low glucose concentration (C-medium) and contact with human fibroblasts (HFF-1) seemed to downregulate the expression of all DNases. *spd1* is the only DNase that is downregulated in the stationary phase and biofilm conditions and upregulated by the presence of serum. The presence of pharyngeal cells (Detroit) upregulates the expression of *spd1* and *sdn* and has an inverse effect on *spd3* and *spd4*. Contact with keratinocytes (HaCaT) upregulates *spd1*, *spd4*, and *sdn*, without affecting *spd3* expression. These data indicate that the four DNases probably have distinct roles in different GAS invasion steps. In parallel, *spd1*, *spd3*, and *spd4* expressions were compared with their expression in the Manfredo strain under the same conditions. We have observed that *spd4* was always more highly expressed in the LO1 strain than in Manfredo, regardless of the tested conditions (Figure 3B). Interestingly, the alignment of *spd4* promoters of the LO1, the three Scottish iGAS, and the Manfredo strains highlighted a point mutation between the predicted −10 and −35 boxes for all but the Manfredo strains (Supplementary Figure S3). No significant differences were observed between *spd1* and *spd3*.

### 3.3. Sdn, Spd1, and Spd3 Have In Vitro Nuclease Activity

We hypothesized that each DNase could have either a distinct role in virulence, specific substrate(s) or range of action (depending on the environment in which the DNase is active). First, we cloned and expressed the four DNases as recombinant proteins with a C-terminal His-tag. Unfortunately, Spd4 was unstable after purification and discarded from further experiments. Point mutations of the catalytic histidine residue were also generated for Sdn (Sdn^H184A^), Spd1 (Spd1^H121A^), and Spd3 (Spd3^H122A^) (Supplementary Figure S4).

**Figure 4:**
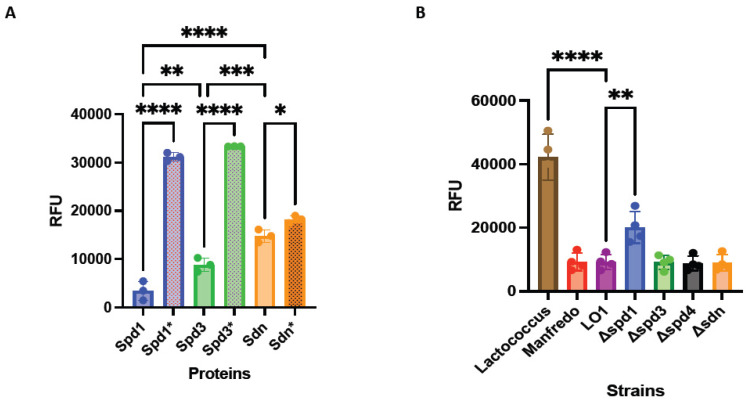
DNase activity of recombinant DNases and their catalytic mutants (*) using Sytox Orange-labeled DNA (**A**). DNase activity in strains supernatants using Sytox Orange-labeled DNA. Lactococcus supernatants were used as negative control (**B**). RFU: relative fluorescent unit. The statistical test used is the one-way ANOVA (*n* = 4 for supernatants; *n* = 3 for recombinant proteins). * p < 0.05, ** *p* < 0.01, *** *p* < 0.001 and **** *p* < 0.0001.

We first performed qualitative in vitro degradation of plasmid DNA at different temperatures representative of potential environments encountered by GAS (28 °C for throat/skin surfaces, 37 °C for physiological temperature, and 40 °C for high fever) and different pH (pH 5, which can be found in necrotizing soft tissue infection [53] or in phagosomes [54], and pH 7, physiological pH) (Supplementary Figure S5). We then tested the degradation of different nucleic acid matrices, that is, genomic bacterial DNA, human genomic DNA, human RNA, and bacterial RNA under physiological conditions (37 °C, pH 7). We found that Sdn and Spd3 were active on all substrates, under all the conditions tested (Supplementary Figures S5 and S6). In contrast, Spd1 was found to be less active at 28 °C, degraded only partially GAS RNA, but did not degrade human RNA. The mutation of the histidine residue in the catalytic site of each DNase abrogated, or at least decreased, nuclease activity (Supplementary Figures S5 and S6). Second, we quantified the in vitro degradation of plasmid DNA at 37 °C using SYTOX Orange assay (Figure 4A). Overall, Spd1 seems to be more active than the others, and Sdn can work under every condition and on each substrate tested.

Knock-out (KO) mutants of DNase (Δ*spd1*, Δs*pd3*, Δ*spd4*, and Δ*sdn*) were constructed. The DNA degrading activity of the LO1, Manfredo, and the four KO mutant supernatants was also tested by SYTOX Orange assay. We observed that the DNase activity was similar between Manfredo and LO1 supernatants (Figure 4B). The same was observed between the LO1 and Δs*pd3*, Δ*spd4*, and Δ*sdn* supernatants. Only Δ*spd1* mutant showed a decrease in DNase activity, confirming that Spd1 is the most active DNase in the LO1 strain (Figure 4B). However, Spd1 is not the only active DNase given that the Δ*spd1* strain still possesses DNase activity.

### 3.4. The DNase KO Mutants Are Not Impaired in Biofilm Formation In Vitro

DNases are known to be important for bacterial metabolism, for example, by providing nucleotide precursors in starvation conditions, such as those encountered in serum or in deep tissues [55]. Therefore, we monitored the growth of the four knock-out mutants (Δ*spd1*, Δs*pd3*, Δ*spd4*, and Δ*sdn*) in comparison with the WT LO1 and Manfredo strains in different media: a glucose rich medium (THY 0.5%), a medium that mimics necrotic conditions, rich in peptides, and poor in sugar (C-medium [24]), and in human serum, where nucleotide precursors are scarce (100% or 20% to mimic blood and mucosa, respectively). No growth differences were observed between the LO1 mutant and WT strains under all tested conditions (Supplementary Figure S7). However, the Manfredo strain showed a slower growth rate and a lower OD_max_ under THY and C-medium conditions compared to the LO1 strain (Supplementary Figure S7).

**Figure 5:**
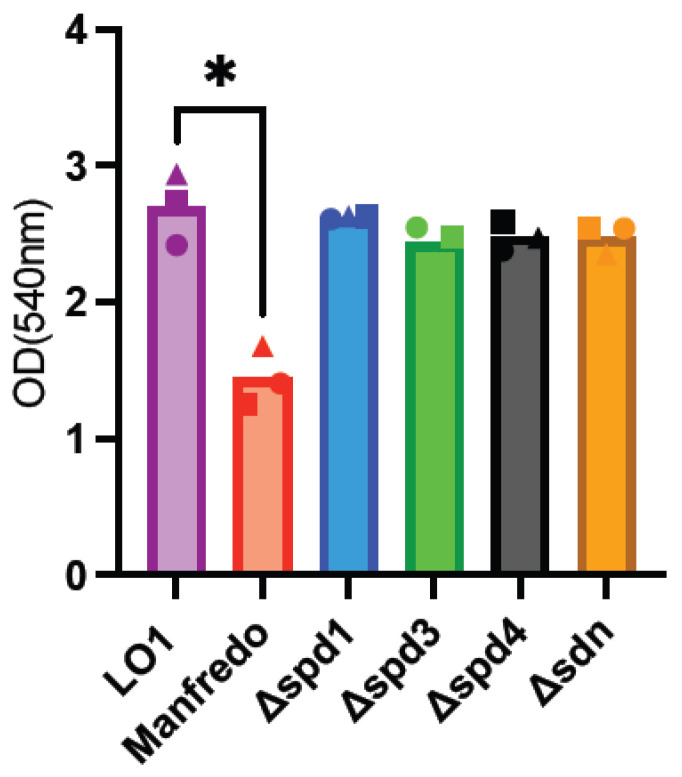
Analysis of biofilm formation by crystal violet staining of the Manfredo, LO1, and its DNase mutants grown on polystyrene plates in THY. The statistical test used is a one-way ANOVA (*n* = 3). * *p* < 0.05.

GAS biofilms are known to be associated with clinical manifestations, notably necrotizing fasciitis [56,57,58]. As LO1 was isolated from a case of nosocomial transmission of necrotizing fasciitis [22], we aimed to compare biofilm formation with the Manfredo strain and the role of DNases in this process. Indeed, the expression of all DNases, except *spd1,* was upregulated in biofilms (Figure 3). Biofilm formation was quantified after 72 h using crystal violet staining [35]. LO1 and its derivatives were found to form higher amounts of biofilms than Manfredo in THY (Figure 5), but the DNases did not appear to be involved in biofilm formation and/or regulation under our in vitro conditions.

### 3.5. The DNase KO Mutants Adhere to HaCaT and Detroit Cells Like the WT Strain

**Figure 6:**
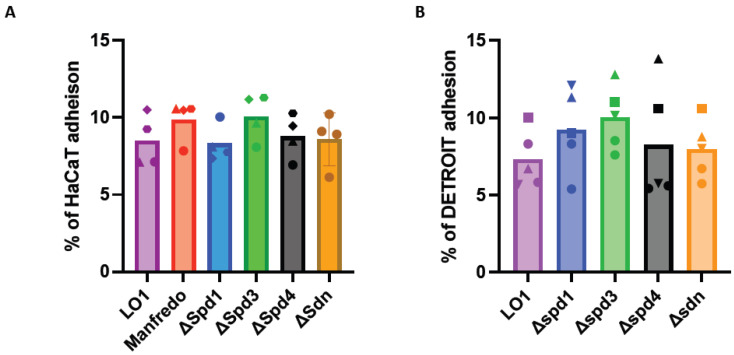
Adhesion of the DNase mutants to HaCaT (**A**) (*n* = 4) or Detroit (**B**) (*n* = 3) cell lines compared to the LO1 strain. The results are presented as a percentage of adhesion in comparison to the initial inoculum. The statistical test used is a one-way ANOVA.

Since contact with HaCaT and Detroit cells seemed to have a positive effect on the expression of some DNases (Figure 3), we decided to study the adhesion of these mutants. After growing the LO1, Manfredo, and DNase mutants to the mid-exponential phase, we infected human cell lines encountered by GAS during colonization. All the tested strains were found to adhere to the two different cell lines (HaCaT cells (keratinocytes) and Detroit cells (pharyngeal epithelial cells), but no difference was observed between the Manfredo, LO1, and DNase mutants (Figure 6).

### 3.6. Spd1, Spd4, and Sdn Are Involved in Whole Blood Survival

**Figure 7:**
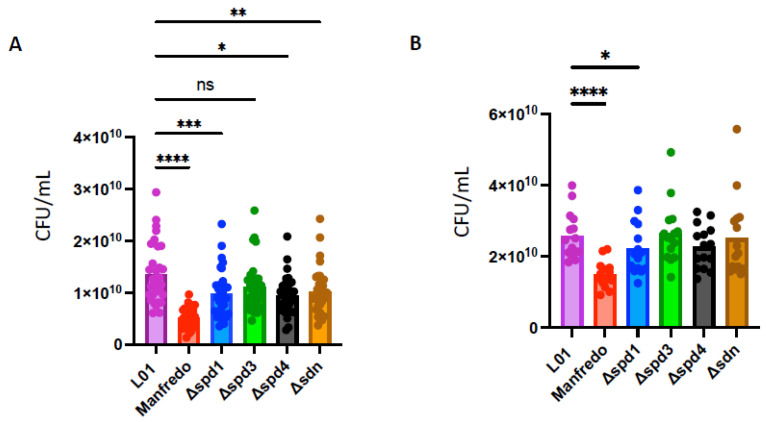
Whole blood survival of the WT Manfredo, LO1, and DNases knock-out mutants. Colony-forming unit (CFU) count after 3 h in whole blood from 6 non-immune donors in absence (**A**) or in presence of cytochalasin D (**B**). The statistical test used is a non-parametric Friedman’s ANOVA test (*n* = 4). ns: non-significant, * *p* < 0.05, ** *p* < 0.01, *** *p* < 0.001 and **** *p* < 0.0001.

As DNases can prevent bacterial killing by evading the immune system of the host [16,19], we tested the survival of DNase mutants in human blood and compared them to the LO1 and Manfredo strains. We performed a whole blood killing assay [59], with blood from six non-immune donors to the M5 strains. To determine the specific effect of DNases on NET degradation, we used cytochalasin D to prevent phagocytosis-mediated killing. After 3 h of incubation, we found that the LO1 strain survived better in whole blood than the Manfredo strain, in both conditions tested (Figure 7A,B). We also observed that *spd1*, *spd4*, and *sdn* mutants survived less than the WT strain in untreated blood, to different extents (Figure 7A). Interestingly, in the presence of cytochalasin D, only the *spd1* mutant showed impaired survival, suggesting a role in NET degradation (Figure 7B).

## 4. Discussion

The LO1 iGAS strain was isolated from a rare case of nosocomial transmission of necrotizing fasciitis and seems to represent an interesting opportunity to understand the virulence of the M5 strain. Interestingly, Pagnossin et al. reported three cases of M5 iGAS in Scotland [38]. Analysis of the chromosomal-encoded virulence factors in the four iGAS and the M5 historical strain (Manfredo) highlighted that *sclA* (encoding streptococcal collagen-like surface protein A) presents a premature stop codon in the four iGAS. Interestingly, a nonsense mutation in *sclA* has been described in invasive M3 isolates [60]. These strains express a truncated protein with a premature stop codon in the GXY repeats which contributes to their invasiveness [61,62,63] (Supplementary Figure S8). Additionally, we found that the four iGAS displayed a different *sclB* gene (streptococcal collagen-like surface protein B) than the Manfredo strain. The impact of sequence differences in SclA/B on biofilm formation should be investigated, as the LO1 strain exhibits greater biofilm formation than the Manfredo strain. Finally, unlike several invasive isolates [64], no mutations in the CovR/S TCS were observed in LO1. However, mutations in YehU and FasC (Supplementary Figure S1) have been highlighted, and their potential roles in virulence require further investigation.

The comparison of the LO1 genome with those of the three Scottish isolates and the Manfredo historical strain shows that the major differences reside in genome rearrangements and mobile genetic element (prophages and transposons) content. Interestingly, an asymmetric genome rearrangement has been observed in a hypervirulent M23 strain and is proposed to play a role in virulence by re-clustering a broad set of CovRS-regulated virulence and metabolic genes to the same leading strand [64]. Inversions in Manfredo and iGAS391/iGAS426 did not have a significant impact on the genome integrity (GC skew) or re-clustering of virulence genes (Supplementary Figure S9). The acquisition of mobile genetic elements, like prophages, can lead to emerging clones of GAS responsible for epidemic outbreaks [65,66]. All five strains possess four prophages and one prophage satellite (SpyCIM). However, the ϕMan.F in Manfredo, coding for the superantigen SpeH and a truncated SpeI (premature stop codon at position 82-, due to a frameshift in a poly(T) stretch), is absent from the other strains. In contrast, the four iGAS strains possessed the ϕLO1.A, coding for the DNase and Sdn (Figure 1). Analysis of the 2.927 available genomes in NCBI showed that only seven strains (0.23%) possessed four DNases, among which included LO1 and the three Scottish isolates (Table S4). We also observed that Spd4, Sda1, and Sdn were the rarest DNases found in GAS (Supplementary Figure S2). Recently, Bah et al. observed that isolates from skin infection in low income countries harbored only a few DNases (24% with *spd1*, 2% with *spd3*) in contrast to strains in high-income countries (56% *spd1*, 70% *spd3*, 5% *sda1*, 16% *sda2*, 16% *sdn*, and 7% *spd4*) [67]. Overall, it seems that the association of these four DNases is rare and that strains carrying this association are potentially highly virulent.

Our RNA-seq data and DNase/RNase activity tests suggest that each DNase in the LO1 strain may have a specific role in virulence. Expression data for the LO1 strain showed that all DNases were downregulated in contact with fibroblasts and in C-medium (glucose poor), suggesting no specific role in deep tissue infections. Both *spd1* and *sdn* are upregulated in the presence of Detroit cells, which has been shown previously [68], whereas both *spd3* and *spd4* are downregulated. However, none of the mutants showed a default state in Detroit cell adhesion. Contact with HaCaT cells induced the expression of *spd1*, *sdn*, and *spd4*, but no role of DNases in HaCaT cell adhesion was observed. We did not identify a condition in which the *spd3* was upregulated or a phenotype associated with the absence of *spd3*. DNases may be important for further steps in colonization and are primed by cell contact with a role inside the host–cell or the subjacent tissues. It has been suggested that DNases can digest host DNA to kill cells or clean debris at the site of infection, allowing GAS dissemination. Moreover, cell contact can also induce prophages [69], and the upregulation of DNases genes could also be linked to prophage induction and phage dissemination. However, the expression of prophage-encoded toxins is not always linked to prophage induction [68]. In addition, DNases can prevent the recognition of GAS RNA/DNA in non-phagocytic cells [70,71]. The *spd1* gene is also upregulated in human serum, which can correlate with its well-known role in NET degradation [72], which was confirmed by our survival test in the presence of cytochalasin D. *spd1* is also induced in vivo during upper respiratory infections where neutrophils are the first line of defense [17,69,73,74]. Interestingly, *spd4* was consistently upregulated in the LO1 strain compared to that in the Manfredo strain under all tested conditions, suggesting a constitutively higher expression in the LO1 background. The alignment of the *spd4* promoter of Manfredo, LO1, and the three Scottish iGAS highlights a point mutation close to the −10/−35 boxes. Further experiments are needed to determine whether this point mutation improves *spd4* expression.

Streptococcal DNases may also be involved in managing biofilms because extracellular DNA (eDNA) is an important component of the extracellular polymeric substance (EPS) matrix [75]. Some clinical cases of necrotizing fasciitis can worsen when bacteria establish biofilms at the infection site [76]. One of the major factors in biofilm formation is the M protein [77,78,79]. The ability of GAS to form strong biofilms seems to be *emm*-type dependent, although differences are also observed between strains belonging to the same *emm*-type [80]. DNases are also known to regulate biofilm formation by degrading extracellular DNA. However, none of the DNases were upregulated in biofilms, and none seemed to be implicated in this process. We observed that LO1 formed a higher amount of biofilm than the Manfredo strain in glucose-rich and glucose-poor media. This could be linked to a higher yield and/or growth rate for the LO1 strain compared to that in the Manfredo strain (Supplementary Figure S7). Interestingly, we observed that the *emm5* gene was upregulated by four-fold in LO1 than for the Manfredo strain when they formed biofilms. An in-depth study of the genes expressed in biofilms by both strains could help to understand this difference.

Our site-directed mutagenesis results showed that the enzymatic activity of the three DNases requires the presence of a histidine residue as described for non-specific endonucleases [81]. Recombinant Sdn exhibits broad activity, degrading plasmid DNA and genomic DNA/RNA from GAS and human hosts. Spd3 possesses both RNase and DNase activities, except for GAS genomic DNA, thus excluding its role in TLR9 recognition evasion. These data suggest that Spd3 and Sdn could both have roles inside the cells because they are able to degrade host RNA and DNA. RNase activity against GAS RNA could also act as a post-transcriptional regulatory mechanism and should be investigated further by RNA-seq analysis of KO mutants [82,83]. As already stated by Broudy et al. [69], Spd1 has no RNase activity but seems to be the more efficient DNase in the LO1 supernatant. Spd1 is the only nuclease that is affected by temperature as its activity is inhibited at 28 °C, a temperature found on the skin. Its frequency in GAS genomes (38%) and its presence in a successful strain such as the M1T1 clone, and more recently M1_UK_, suggest an important role in GAS virulence. Finally, we were unable to purify Spd4, even its mutated form, suggesting the need for a chaperone for its stabilization [21].

The *sdn* and *spd4* mutants survived less than the WT strain in the blood but not in the presence of cytochalasin D, suggesting that they are implicated in blood survival independent of NET degradation. DNase secretion in the environment or inside host cells can lead to enhanced resistance against the host immune system by escaping the TLR9 recognition system [16,17,19] or inducing IFN-1 production by dendritic cells at the infection site [84]. Interestingly, the Manfredo strain survived less than the LO1 strain under all the conditions tested. Given that they are from the same *emm*-type and are genetically very close, we postulate that the additional DNase, the increased expression of *spd4* or a specific regulation (via FasBCA or YehUT) in the LO1 strain could explain this phenotype.

## 5. Conclusions

In conclusion, we have shown that the LO1 strain is genetically related to the M5 historical strain Manfredo yet presents distinct virulence phenotypes. We have shown that Spd4 and Sdn play a role in blood survival, even though their exact roles within a human host are not fully understood. In contrast, we found that the DNases of the LO1 strain are not involved in adhesion to host cells or biofilm formation. This study highlights the complex and systemic interplay between different bacterial virulence pathways which complicates deciphering the molecular roots of a given clinical case. Our study is mainly based on genomic comparison and in vitro observations of two M5 strains. The biofilm capacity of several GAS strains should be investigated in depth and in more physiological conditions. Moreover, performing a complete biofilm transcriptomic study would help in our understanding of GAS biology in necrotizing fasciitis.

## Figures and Tables

**Table 1:** Genomic comparison of LO1, iGAS376, iGAS391, iGAS426, and Manfredo.

	iGAS				
Characteristics	iGAS376	iGAS391	iGAS426	LO1	Manfredo
Year of isolation	2018	2019	2015	2010	1952
Origin	Edinburgh, Scotland	Glasgow, Scotland	Glasgow, Scotland	Brussels, Belgium	Chicago, USA
Source	Blood	Blood	Blood	NF	ARF
Genome size (bp)	1,897,124	1,897,129	1,897,111	1,897,626	1,841,271
GC content (%)	38.6	38.6	38.6	38.6	38.6
No. of genes	1913	1913	1913	1928	1841
MLST	ST99	ST99	ST99	ST99	ST99
Numbers of prophages	5	5	5	5	5
Number of transposon	1	1	1	1	0
GenBank accession no.	CP067010	CP067009	CP067008	CP156075	NC_009332
Reference	[38]	[38]	[38]	[22], this study	[23,37]

**Table 2:** Percentage of identity between the virulence factors of the LO1, iGAS376, iGAS391, iGAS426, and Manfredo strains. Proteins that are not 100% identical in sequence are highlighted in orange.

Gene	Locus-Tag		% of Identity with LO1	
		Virulence Factor	Scottish iGAS	Manfredo
*emm5*	*lamri_01793*	M protein	94	93
*ska*	*lamri_01765*	Streptokinase	100	146 aa smaller
*cfa*	*lamri_01071*	CAMP factor	100	100
*slo*	*lamri_00241*	Streptolysin O	100	100
*sagA, B, C, D, E, F, H, I*	*lamri_00692-700*	Streptolysin S	100	100
hylA*	*lamri_00886*	Hyaluronate lyase precursor	100	100
*hasA,B,C*	*lamri_01939, lamri_01940,lamri_1941, lamr_00274, lamri_00523*	Capsule	100	100
*spyCEP*	*lamri_00421*	cysteine protéase	99.9	99.9
*sibA*	*lamri_00049*	cysteine protéase	100	100
*speB*	*lamri_01808*	Cysteine Protease B	100	100
*speC*	*lamri_00674*	Streptococcal pyrogenic exotoxin C *	100	100
*speG*	*lamri_00269*	Streptococcal pyrogenic exotoxin G	100	100
*speK*	*lamri_1644*	Streptococcal pyrogenic exotoxin K	100	100
*smeZ*	*lamri_01783*	Streptococcal mitogenic exotoxin Z	100	100
*scpA*	*lamri_01792*	C5a peptidase	100	100
*endoS*	*lamri_01629*	endo-beta-N-acetylglucosaminidase	100	100
*spnA*	*lamri_00701*	Chromosome-encoded nuclease	100	100
*sdaB*	*lamri_01811*	Chromosome-encoded nuclease	100	100
*nga*	*lamri_00239*	NAD (Spn)	100	100
*isp*	*lamri_01797*	Isp	100	100
*inlA1*	*lamri_01140*	Internalin 1	100	100
*inlA2 (shr)*	*lamri_01622*	Internalin 2	100	100
*fbp*	*lamri_00872*	Fn binding protein	100	99.9
*fba*	*lamri_01691*	Fibronectin binding proteins	100	100
*lmb*	*lmari_01791*	Laminin binding protein	100	100
*cpa/fctX*	*lamri_00209*	cpa/fctX	100	100
*tee/fctA*	*lamri_00211*	tee/fctA	100	100
*prtf2*	*lamri_00215*	Fibronectin binding proteins	100	100
*sclB*	*lamri_00900*	Scl-like	72	67
*sclA*	*lamri_01768*	scl	100	55 aa longer
*grab*	*lamri_01137*	GRAB	99.9	99.9
*hypothetical protein*	*lamri_01261*	LPxTG anchored protein	100	100
*hypothetical protein*	*lamri_00783*	LPxTG anchored protein LRR repeat	100	100
*sdn*	*lamri_00102*	prophage-encoded nuclease	100	Absent
*spd1*	*lamri_00675*	prophage-encoded nuclease	100	100
*spd3*	*lamri_01348*	prophage-encoded nuclease	100	100
*spd4*	*lamri_01201*	prophage-encoded nuclease	100	100

* Internal stop (codon 73) in all strains.

## Data Availability

The data that support the findings of this study are openly available at NCBI, under Bioproject PRJNA1102170 (LO1 genome and RNA-seq data).

## Supplementary Materials

The following supporting information can be downloaded at: https://www.mdpi.com/article/10.3390/microorganisms12112209/s1, Figure S1: Alignment of the TCS FasBCA and YehUT from Manfredo and L01 using Geneious® 2023.2.1. Figure S2: Frequencies of prophages-associated DNase in GAS (n=2.992). Figure S3: Alignment of the promoters of *spd4* from Manfredo, L01 and the 3 Scottish iGAS strains using Geneious Prime®2023.2.1. -10/-35 box was predicted using BPROM [30]. Figure S4: (**A**) Alignment of WT and mutated version of Sdn, Spd and Spd3. The histidine residue in the catalytic site is indicated by the black arrow. In each mutated DNase, it was replaced by an alanine. (**B**) AlphaFold 2 [85,86] prediction of each L01 DNase structure. The catalytic RGH-like motif is highlighted in red and with an arrow (the motif is buried in the Spd3 structure). Figure S5: Agarose gels separation of plasmidic DNA incubated with the purified Spd1, Spd3 and Sdn as well as their mutated counterpart (Sdn^H184A^, Spd1^H121A^, Spd3^H122A^) (**A**) at 37°C, pH5 and pH7 (**B**) at 28° and 40°C at pH7. AB: activity buffer, AB: elution buffer. MW: molecular weight. Figure S6: Agarose gel electrophoresis of (**A**) genomic human DNA (_H_DNA), (**B**) genomic GAS DNA (_G_DNA) or (**C**) human (_H_RNA) and GAS RNA (_G_RNA) incubated with the purified Spd1, Spd3 and Sdn as well as their catalytic mutant (Sdn^H184A^, Spd1^H121A^, Spd3^H122A^) at 37°C and pH 7. The DNaseI or RNase A have been used as positive control. AB: activity buffer, MW: molecular weight. Figure S7: Growth curves of the Manfredo strain, the L01 and its mutants in (**A**) THY, (**B**) C-media, (**C**) serum 20% or (**D**) 100 %. Figure S8: Alignment of (**A**) SclA and (**B**) SclB from Manfredo, L01 and SSI-1 (M3 iGAS) using Geneious Prime® 2023.2.1. Figure S9: (**A**) GC skew of the Manfredo, L01 and the 3 Scottish iGAS genomes using GenSkew [87]. (**B**) Orientation of the virulence genes in the Manfredo, L01 and iGAS391 strains. Figure S10: Whole cell binding of L01 and Manfredo strains in human serum. (**A**) SDS-PAGE and Coomassie blue staining of eluted serum proteins from L01 or Manfredo (**B**) immuno-detection of eluted serum proteins from L01 or Manfredo with anti-albumin, anti-fribrinogen, IgG and alpha-2-macroglubulin. Table S1: Frequencies of prophages-associated DNase in GAS (n=2.992). Table S2: Conditions used for the RNA-sequencing and their characteristics. Table S3: Primers used in this study. Table S4: Strains containing 4 DNases encoding genes among the 2992 available genomes on NCBI.

## References

[1] Walker M.J., Barnett T.C., McArthur J.D., Cole J.N., Gillen C.M., Henningham A., Sriprakash K.S., Sanderson-Smith M.L., Nizet V. (2014). Disease manifestations and pathogenic mechanisms of group a *Streptococcus*. Clin. Microbiol. Rev..

[2] Brouwer S., Rivera-Hernandez T., Curren B.F., Harbison-Price N., De Oliveira D.M.P., Jespersen M.G., Davies M.R., Walker M.J. (2023). Pathogenesis, epidemiology and control of Group A *Streptococcus* infection. Nat. Rev. Microbiol..

[3] Carapetis J.R., Steer A.C., Mulholland E.K., Weber M. (2005). The global burden of group A streptococcal diseases. Lancet Infect. Dis..

[4] Bessen D.E. (2016). Tissue tropisms in group A *Streptococcus*: What virulence factors distinguish pharyngitis from impetigo strains?. Curr. Opin. Infect. Dis..

[5] Schiavolin L., Deneubourg G., Steinmetz J., Smeesters P.R., Botteaux A. (2024). Group A *Streptococcus* adaptation to diverse niches: Lessons from transcriptomic studies. Crit. Rev. Microbiol..

[6] Smeesters P.R., McMillan D.J., Sriprakash K.S. (2010). The streptococcal M protein: A highly versatile molecule. Trends Microbiol..

[7] Smeesters P.R., Mardulyn P., Vergison A., Leplae R., Van Melderen L. (2008). Genetic diversity of Group A *Streptococcus* M protein: Implications for typing and vaccine development. Vaccine.

[8] Smeesters P.R., de Crombrugghe G., Tsoi S.K., Leclercq C., Baker C., Osowicki J., Verhoeven C., Botteaux A., Steer A.C. (2024). Systematic review of global *Streptococcus* pyogenes strain diversity, disease associations, and implications for vaccine development. Lancet Microbe.

[9] Giovanetti E., Brenciani A., Vecchi M., Manzin A., Varaldo P.E. (2005). Prophage association of mef(A) elements encoding efflux-mediated erythromycin resistance in *Streptococcus* pyogenes. J. Antimicrob. Chemother..

[10] Green N.M., Beres S.B., Graviss E.A., Allison J.E., McGeer A.J., Vuopio-Varkila J., LeFebvre R.B., Musser J.M. (2005). Genetic diversity among type emm28 group A *Streptococcus* strains causing invasive infections and pharyngitis. J. Clin. Microbiol..

[11] Al-Shahib A., Underwood A., Afshar B., Turner C.E., Lamagni T., Sriskandan S., Efstratiou A. (2016). Emergence of a novel lineage containing a prophage in emm/M3 group A *Streptococcus* associated with upsurge in invasive disease in the UK. Microb. Genom..

[12] Lynskey N.N., Jauneikaite E., Li H.K., Zhi X., Turner C.E., Mosavie M., Pearson M., Asai M., Lobkowicz L., Chow J.Y. (2019). Emergence of dominant toxigenic M1T1 *Streptococcus* pyogenes clone during increased scarlet fever activity in England: A population-based molecular epidemiological study. Lancet Infect. Dis..

[13] Davies M.R., Keller N., Brouwer S., Jespersen M.G., Cork A.J., Hayes A.J., Pitt M.E., De Oliveira D.M.P., Harbison-Price N., Bertolla O.M. (2023). Detection of *Streptococcus* pyogenes M1(UK) in Australia and characterization of the mutation driving enhanced expression of superantigen SpeA. Nat. Commun..

[14] Remmington A., Turner C. (2018). The DNases of pathogenic Lancefield streptococci. Microbiology.

[15] Walker M.J., Hollands A., Sanderson-Smith M.L., Cole J.N., Kirk J.K., Henningham A., McArthur J.D., Dinkla K., Aziz R.K., Kansal R.G. (2007). DNase Sda1 provides selection pressure for a switch to invasive group A streptococcal infection. Nat. Med..

[16] Buchanan J.T., Simpson A.J., Aziz R.K., Liu G.Y., Kristian S.A., Kotb M., Feramisco J., Nizet V. (2006). DNase expression allows the pathogen group A *Streptococcus* to escape killing in neutrophil extracellular traps. Curr. Biol..

[17] Sumby P., Barbian K.D., Gardner D.J., Whitney A.R., Welty D.M., Long R.D., Bailey J.R., Parnell M.J., Hoe N.P., Adams G.G. (2005). Extracellular deoxyribonuclease made by group A *Streptococcus* assists pathogenesis by enhancing evasion of the innate immune response. Proc. Natl. Acad. Sci. USA.

[18] Chang A., Khemlani A., Kang H., Proft T. (2011). Functional analysis of *Streptococcus* pyogenes nuclease A (SpnA), a novel group A streptococcal virulence factor. Mol. Microbiol..

[19] Uchiyama S., Andreoni F., Schuepbach R.A., Nizet V., Zinkernagel A.S. (2012). DNase Sda1 allows invasive M1T1 Group A *Streptococcus* to prevent TLR9-dependent recognition. PLoS Pathog..

[20] Korczynska J.E., Turkenburg J.P., Taylor E.J. (2012). The structural characterization of a prophage-encoded extracellular DNase from *Streptococcus* pyogenes. Nucleic Acids Res..

[21] Moon A.F., Krahn J.M., Lu X., Cuneo M.J., Pedersen L.C. (2016). Structural characterization of the virulence factor Sda1 nuclease from *Streptococcus* pyogenes. Nucleic Acids Res..

[22] Sablier F., Slaouti T., Drèze P.A., El Fouly P.E., Allemeersch D., Van Melderen L., Smeesters P.R. (2010). Nosocomial transmission of necrotising fasciitis. Lancet.

[23] Holden M.T., Scott A., Cherevach I., Chillingworth T., Churcher C., Cronin A., Dowd L., Feltwell T., Hamlin N., Holroyd S. (2007). Complete genome of acute rheumatic fever-associated serotype M5 *Streptococcus* pyogenes strain manfredo. J. Bacteriol..

[24] Gera K., McIver K.S. (2013). Laboratory growth and maintenance of *Streptococcus* pyogenes (the Group A *Streptococcus*, GAS). Curr. Protoc. Microbiol..

[25] Davies M.R., McIntyre L., Mutreja A., Lacey J.A., Lees J.A., Towers R.J., Duchene S., Smeesters P.R., Frost H.R., Price D.J. (2019). Atlas of group A streptococcal vaccine candidates compiled using large-scale comparative genomics. Nat. Genet..

[26] Tanizawa Y., Fujisawa T., Nakamura Y. (2018). DFAST: A flexible prokaryotic genome annotation pipeline for faster genome publication. Bioinformatics.

[27] McNeil L.K., Reich C., Aziz R.K., Bartels D., Cohoon M., Disz T., Edwards R.A., Gerdes S., Hwang K., Kubal M. (2007). The National Microbial Pathogen Database Resource (NMPDR): A genomics platform based on subsystem annotation. Nucleic Acids Res..

[28] Jolley K.A., Bray J.E., Maiden M.C.J. (2018). Open-access bacterial population genomics: BIGSdb software, the PubMLST.org website and their applications. Wellcome Open Res..

[29] Darling A.E., Mau B., Perna N.T. (2010). progressiveMauve: Multiple genome alignment with gene gain, loss and rearrangement. PLoS ONE.

[30] Solovyev V.S.A., Li R.W. (2011). Automatic Annotation of Microbial Genomes and Metagenomic Sequences. Metagenomics and Its Applications in Agriculture, Biomedicine and Environmental Studies.

[31] Alikhan N.F., Petty N.K., Ben Zakour N.L., Beatson S.A. (2011). BLAST Ring Image Generator (BRIG): Simple prokaryote genome comparisons. BMC Genom..

[32] Le Breton Y., McIver K.S. (2013). Genetic manipulation of *Streptococcus* pyogenes (the Group A Streptococcus, GAS). Curr. Protoc. Microbiol..

[33] Barnett T.C., Daw J.N., Walker M.J., Brouwer S. (2020). Genetic Manipulation of Group A *Streptococcus*-Gene Deletion by Allelic Replacement. Methods Mol. Biol..

[34] Schiavolin L., Lakhloufi D., Botquin G., Deneubourg G., Bruyns C., Steinmetz J., Henrot C., Delforge V., Smeesters P.R., Botteaux A. (2024). Efficient and rapid one-step method to generate gene deletions in *Streptococcus* pyogenes. Microbiol. Spectr..

[35] Vyas H.K.N., McArthur J.D., Sanderson-Smith M.L. (2021). An optimised GAS-pharyngeal cell biofilm model. Sci. Rep..

[36] Aranha M.P., Penfound T.A., Salehi S., Botteaux A., Smeesters P., Dale J.B., Smith J.C. (2021). Design of Broadly Cross-Reactive M Protein-Based Group A Streptococcal Vaccines. J. Immunol..

[37] Siegel A., Johnson E., Stollerman G. (1961). Controlled studies of streptococcal pharyngitis in a pediatric population. 1. Factors related to the attack rate of rheumatic fever. N. Engl. J. Med..

[38] Pagnossin D., Smith A., Weir W., Crestani C., Lindsay D., Ure R., Oravcova K. (2021). Complete Genome Sequences of Three Invasive Strains of *Streptococcus* pyogenes Subtype emm5.23 Isolated in Scotland. Microbiol. Resour. Announc..

[39] Blagden S., Watts V., Verlander N.Q., Pegorie M. (2020). Invasive group A streptococcal infections in North West England: Epidemiology, risk factors and fatal infection. Public Health.

[40] Degala S., Puleston R., Bates R., Borges-Stewart R., Coelho J., Kapatai G., Chalker V., Mair-Jenkins J. (2020). A protracted iGAS outbreak in a long-term care facility 2014-2015: Control measures and the use of whole-genome sequencing. J. Hosp. Infect..

[41] Banks D.J., Beres S.B., Musser J.M. (2002). The fundamental contribution of phages to GAS evolution, genome diversification and strain emergence. Trends Microbiol..

[42] Arndt D., Grant J.R., Marcu A., Sajed T., Pon A., Liang Y., Wishart D.S. (2016). PHASTER: A better, faster version of the PHAST phage search tool. Nucleic Acids Res..

[43] Roberts A.P., Mullany P. (2009). A modular master on the move: The Tn916 family of mobile genetic elements. Trends Microbiol..

[44] McShan W.M., Nguyen S.V., Ferretti J.J., Stevens D.L., Fischetti V.A. (2022). The Bacteriophages of *Streptococcus* pyogenes. Streptococcus Pyogenes: Basic Biology to Clinical Manifestations.

[45] Rezaei Javan R., Ramos-Sevillano E., Akter A., Brown J., Brueggemann A.B. (2019). Prophages and satellite prophages are widespread in *Streptococcus* and may play a role in pneumococcal pathogenesis. Nat. Commun..

[46] Hendrickson C., Euler C.W., Nguyen S.V., Rahman M., McCullor K.A., King C.J., Fischetti V.A., McShan W.M. (2015). Elimination of Chromosomal Island SpyCIM1 from *Streptococcus* pyogenes Strain SF370 Reverses the Mutator Phenotype and Alters Global Transcription. PLoS ONE.

[47] Graham M.R., Smoot L.M., Migliaccio C.A., Virtaneva K., Sturdevant D.E., Porcella S.F., Federle M.J., Adams G.J., Scott J.R., Musser J.M. (2002). Virulence control in group A *Streptococcus* by a two-component gene regulatory system: Global expression profiling and in vivo infection modeling. Proc. Natl. Acad. Sci. USA.

[48] Tran-Winkler H.J., Love J.F., Gryllos I., Wessels M.R. (2011). Signal transduction through CsrRS confers an invasive phenotype in group A *Streptococcus*. PLoS Pathog..

[49] Sumby P., Whitney A.R., Graviss E.A., DeLeo F.R., Musser J.M. (2006). Genome-wide analysis of group a streptococci reveals a mutation that modulates global phenotype and disease specificity. PLoS Pathog..

[50] Buckley S.J., Timms P., Davies M.R., McMillan D.J. (2018). In silico characterisation of the two-component system regulators of *Streptococcus* pyogenes. PLoS ONE.

[51] Siemens N., Lutticken R. (2021). *Streptococcus* pyogenes (“Group A *Streptococcus*”), a Highly Adapted Human Pathogen-Potential Implications of Its Virulence Regulation for Epidemiology and Disease Management. Pathogens.

[52] Riddle D.J., Bessen D.E., Caparon M.G. (2010). Variation in *Streptococcus* pyogenes NAD+ glycohydrolase is associated with tissue tropism. J. Bacteriol..

[53] Hakkarainen T.W., Kopari N.M., Pham T.N., Evans H.L. (2014). Necrotizing soft tissue infections: Review and current concepts in treatment, systems of care, and outcomes. Curr. Probl. Surg..

[54] Westman J., Grinstein S. (2020). Determinants of Phagosomal pH During Host-Pathogen Interactions. Front. Cell Dev. Biol..

[55] Samant S., Lee H., Ghassemi M., Chen J., Cook J.L., Mankin A.S., Neyfakh A.A. (2008). Nucleotide biosynthesis is critical for growth of bacteria in human blood. PLoS Pathog..

[56] Young C., Holder R.C., Dubois L., Reid S.D., Ferretti J.J., Stevens D.L., Fischetti V.A. (2016). Streptococcus pyogenes Biofilm. Streptococcus Pyogenes: Basic Biology to Clinical Manifestations.

[57] Fiedler T., Köller T., Kreikemeyer B. (2015). *Streptococcus* pyogenes biofilms-formation, biology, and clinical relevance. Front. Cell Infect. Microbiol..

[58] Ogawa T., Terao Y., Okuni H., Ninomiya K., Sakata H., Ikebe K., Maeda Y., Kawabata S. (2011). Biofilm formation or internalization into epithelial cells enable *Streptococcus* pyogenes to evade antibiotic eradication in patients with pharyngitis. Microb. Pathog..

[59] Reglinski M. (2020). Lancefield Whole Blood Killing Assay to Evaluate Vaccine Efficacy. Methods Mol. Biol..

[60] Nakagawa I., Kurokawa K., Yamashita A., Nakata M., Tomiyasu Y., Okahashi N., Kawabata S., Yamazaki K., Shiba T., Yasunaga T. (2003). Genome sequence of an M3 strain of *Streptococcus* pyogenes reveals a large-scale genomic rearrangement in invasive strains and new insights into phage evolution. Genome Res..

[61] Flores A.R., Jewell B.E., Versalovic E.M., Olsen R.J., Bachert B.A., Lukomski S., Musser J.M. (2015). Natural variant of collagen-like protein a in serotype M3 group a *Streptococcus* increases adherence and decreases invasive potential. Infect. Immun..

[62] Shea P.R., Beres S.B., Flores A.R., Ewbank A.L., Gonzalez-Lugo J.H., Martagon-Rosado A.J., Martinez-Gutierrez J.C., Rehman H.A., Serrano-Gonzalez M., Fittipaldi N. (2011). Distinct signatures of diversifying selection revealed by genome analysis of respiratory tract and invasive bacterial populations. Proc. Natl. Acad. Sci. USA.

[63] Oliver-Kozup H.A., Elliott M., Bachert B.A., Martin K.H., Reid S.D., Schwegler-Berry D.E., Green B.J., Lukomski S. (2011). The streptococcal collagen-like protein-1 (Scl1) is a significant determinant for biofilm formation by group A *Streptococcus*. BMC Microbiol..

[64] Bao Y.J., Liang Z., Mayfield J.A., McShan W.M., Lee S.W., Ploplis V.A., Castellino F.J. (2016). Novel genomic rearrangements mediated by multiple genetic elements in *Streptococcus* pyogenes M23ND confer potential for evolutionary persistence. Microbiology.

[65] Davies M.R., Holden M.T., Coupland P., Chen J.H., Venturini C., Barnett T.C., Zakour N.L., Tse H., Dougan G., Yuen K.Y. (2015). Emergence of scarlet fever *Streptococcus* pyogenes emm12 clones in Hong Kong is associated with toxin acquisition and multidrug resistance. Nat. Genet..

[66] Afshar B., Turner C.E., Lamagni T.L., Smith K.C., Al-Shahib A., Underwood A., Holden M.T.G., Efstratiou A., Sriskandan S. (2017). Enhanced nasopharyngeal infection and shedding associated with an epidemic lineage of emm3 group A *Streptococcus*. Virulence.

[67] Bah S.Y., Keeley A.J., Armitage E.P., Khalid H., Chaudhuri R.R., Senghore E., Manneh J., Tilley L., Marks M., Darboe S. (2023). Genomic Characterization of Skin and Soft Tissue *Streptococcus* pyogenes Isolates from a Low-Income and a High-Income Setting. mSphere.

[68] Banks D.J., Lei B., Musser J.M. (2003). Prophage induction and expression of prophage-encoded virulence factors in group A *Streptococcus* serotype M3 strain MGAS315. Infect. Immun..

[69] Broudy T.B., Pancholi V., Fischetti V.A. (2002). The in vitro interaction of *Streptococcus* pyogenes with human pharyngeal cells induces a phage-encoded extracellular DNase. Infect. Immun..

[70] Schmolke M., Patel J.R., de Castro E., Sanchez-Aparicio M.T., Uccellini M.B., Miller J.C., Manicassamy B., Satoh T., Kawai T., Akira S. (2014). RIG-I detects mRNA of intracellular Salmonella enterica serovar Typhimurium during bacterial infection. mBio.

[71] Mohamed W., Domann E., Chakraborty T., Mannala G., Lips K.S., Heiss C., Schnettler R., Alt V. (2016). TLR9 mediates S. aureus killing inside osteoblasts via induction of oxidative stress. BMC Microbiol..

[72] Brouwer S., Barnett T.C., Ly D., Kasper K.J., De Oliveira D.M.P., Rivera-Hernandez T., Cork A.J., McIntyre L., Jespersen M.G., Richter J. (2020). Prophage exotoxins enhance colonization fitness in epidemic scarlet fever-causing *Streptococcus* pyogenes. Nat. Commun..

[73] Broudy T.B., Pancholi V., Fischetti V.A. (2001). Induction of lysogenic bacteriophage and phage-associated toxin from group a streptococci during coculture with human pharyngeal cells. Infect. Immun..

[74] Unnikrishnan M., Altmann D.M., Proft T., Wahid F., Cohen J., Fraser J.D., Sriskandan S. (2002). The bacterial superantigen streptococcal mitogenic exotoxin Z is the major immunoactive agent of *Streptococcus* pyogenes. J. Immunol..

[75] Sharma K., Pagedar Singh A. (2018). Antibiofilm Effect of DNase against Single and Mixed Species Biofilm. Foods.

[76] Siemens N., Chakrakodi B., Shambat S.M., Morgan M., Bergsten H., Hyldegaard O., Skrede S., Arnell P., Madsen M.B., Johansson L. (2016). Biofilm in group A streptococcal necrotizing soft tissue infections. JCI Insight.

[77] Courtney H.S., Ofek I., Penfound T., Nizet V., Pence M.A., Kreikemeyer B., Podbielski A., Hasty D.L., Dale J.B. (2009). Relationship between expression of the family of M proteins and lipoteichoic acid to hydrophobicity and biofilm formation in *Streptococcus* pyogenes. PLoS ONE.

[78] Lembke C., Podbielski A., Hidalgo-Grass C., Jonas L., Hanski E., Kreikemeyer B. (2006). Characterization of biofilm formation by clinically relevant serotypes of group A streptococci. Appl. Environ. Microbiol..

[79] Cho K.H., Caparon M.G. (2005). Patterns of virulence gene expression differ between biofilm and tissue communities of *Streptococcus* pyogenes. Mol. Microbiol..

[80] Baldassarri L., Creti R., Recchia S., Imperi M., Facinelli B., Giovanetti E., Pataracchia M., Alfarone G., Orefici G. (2006). Therapeutic failures of antibiotics used to treat macrolide-susceptible *Streptococcus* pyogenes infections may be due to biofilm formation. J. Clin. Microbiol..

[81] Wu S.L., Li C.C., Chen J.C., Chen Y.J., Lin C.T., Ho T.Y., Hsiang C.Y. (2009). Mutagenesis identifies the critical amino acid residues of human endonuclease G involved in catalysis, magnesium coordination, and substrate specificity. J. Biomed. Sci..

[82] Bugrysheva J.V., Scott J.R. (2010). Regulation of virulence gene expression in *Streptococcus* pyogenes: Determinants of differential mRNA decay. RNA Biol..

[83] Bugrysheva J.V., Scott J.R. (2010). The ribonucleases J1 and J2 are essential for growth and have independent roles in mRNA decay in *Streptococcus* pyogenes. Mol. Microbiol..

[84] Keller N., Woytschak J., Heeb L.E.M., Marques Maggio E., Mairpady Shambat S., Snall J., Hyldegaard O., Boyman O., Norrby-Teglund A., Zinkernagel A.S. (2019). Group A Streptococcal DNase Sda1 Impairs Plasmacytoid Dendritic Cells’ Type 1 Interferon Response. J. Investig. Dermatol..

[85] Jumper J., Evans R., Pritzel A., Green T., Figurnov M., Ronneberger O., Tunyasuvunakool K., Bates R., Žídek A., Potapenko A. (2021). Highly accurate protein structure prediction with AlphaFold. Nature.

[86] Varadi M., Bertoni D., Magana P., Paramval U., Pidruchna I., Radhakrishnan M., Tsenkov M., Nair S., Mirdita M., Yeo J. (2024). AlphaFold Protein Structure Database in 2024: Providing structure coverage for over 214 million protein sequences. Nucleic Acids Res..

[87] Lu J., Salzberg S.L. (2020). SkewIT: The Skew Index Test for large-scale GC Skew analysis of bacterial genomes. PLoS Comput. Biol..

