# Supplementary material for: Nosocomial Transmission of Necrotizing Fasciitis: A Molecular Characterization of Group A Streptococcal DNases in Clinical Virulence
Source: Microorganisms. 2024 Oct 31;12(11):2209. doi: 10.3390/microorganisms12112209 (PMC11596691; doi:10.3390/microorganisms12112209)
Supplement: Supplementary file 1 [file microorganisms-12-02209-s001.zip › Figure Supp .pdf]

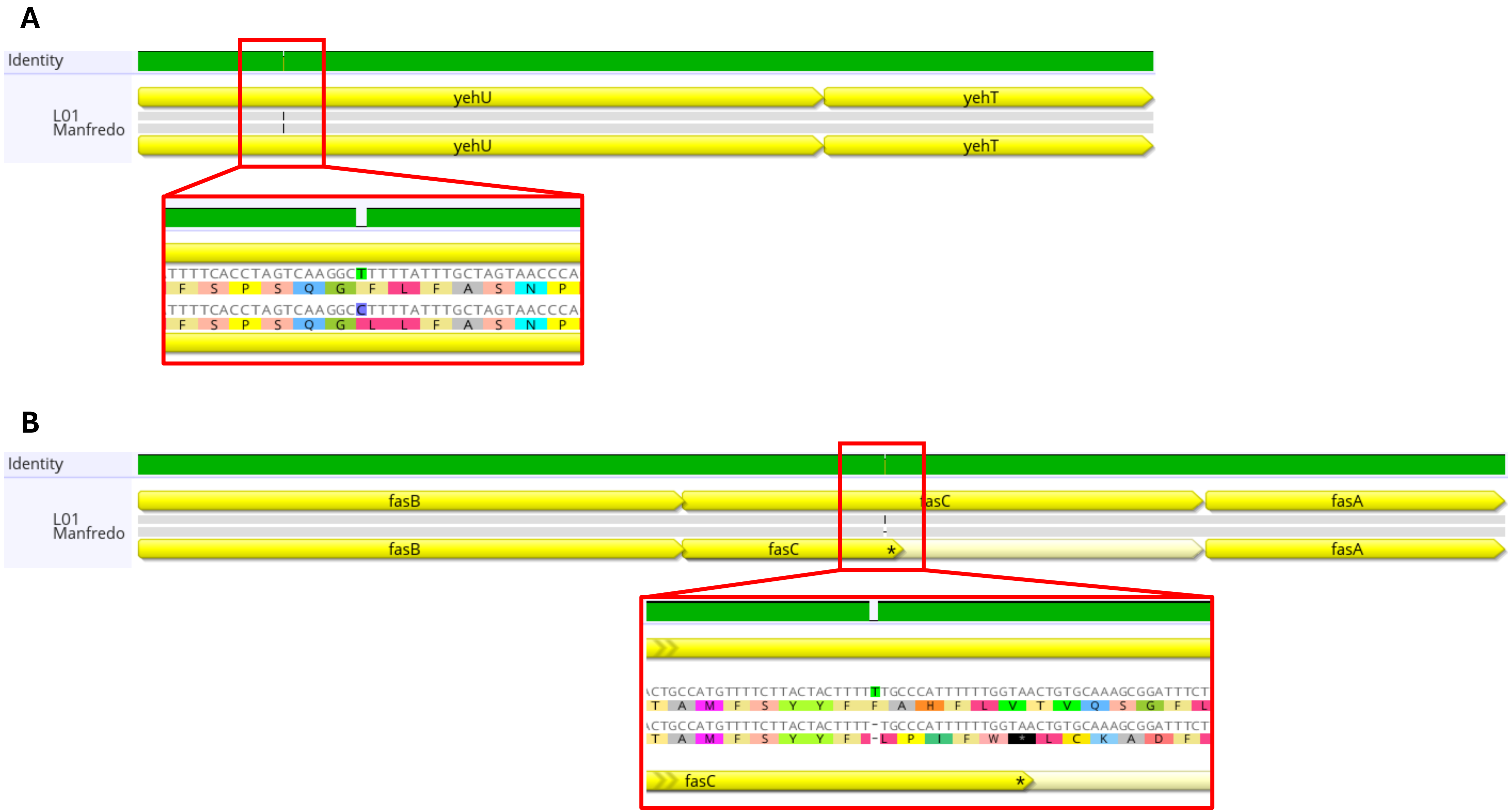

Fig.S1. Alignment of the YehUT (**A**) and FasBCA (**B**) TCS from Manfredo and LO1 using Geneious® 2023.2.1.

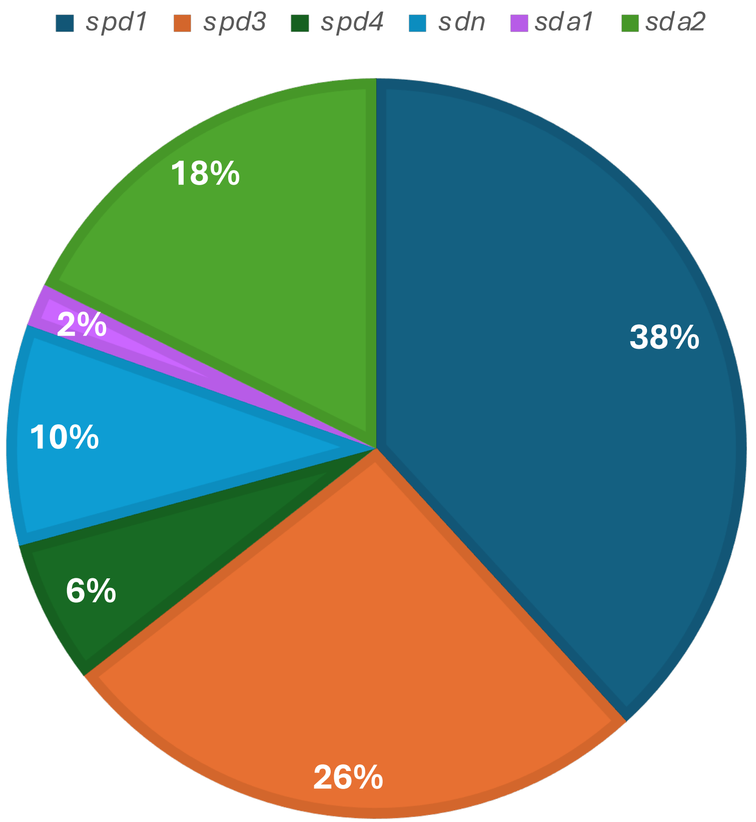

Fig.S2. Frequencies for prophages-associated DNase in GAS (n=2.992).

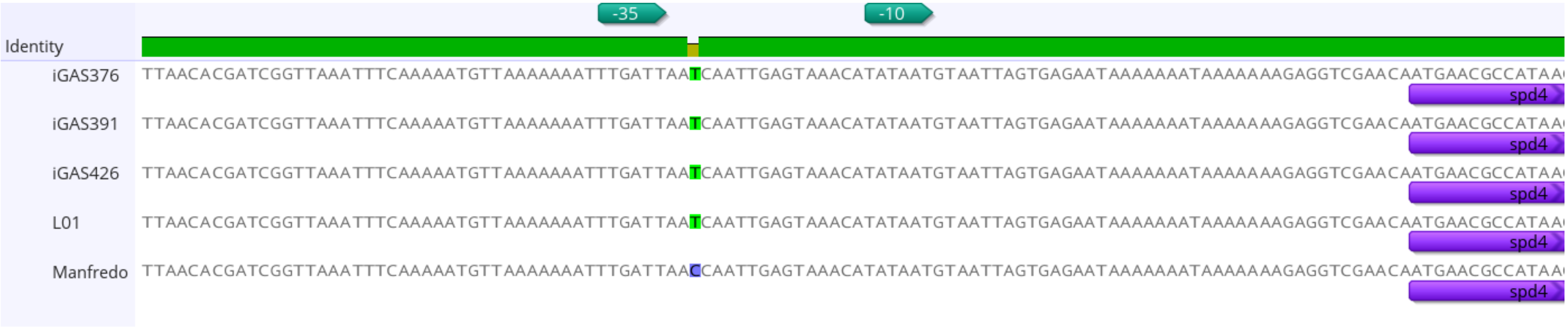

Fig.S3. Alignment of the promoters of *spd4* from Manfredo, LO1 and the 3 Scottish iGAS strains using Geneious Prime®2023.2.1. -10/-35 boxes were predicted using BPROM [30].

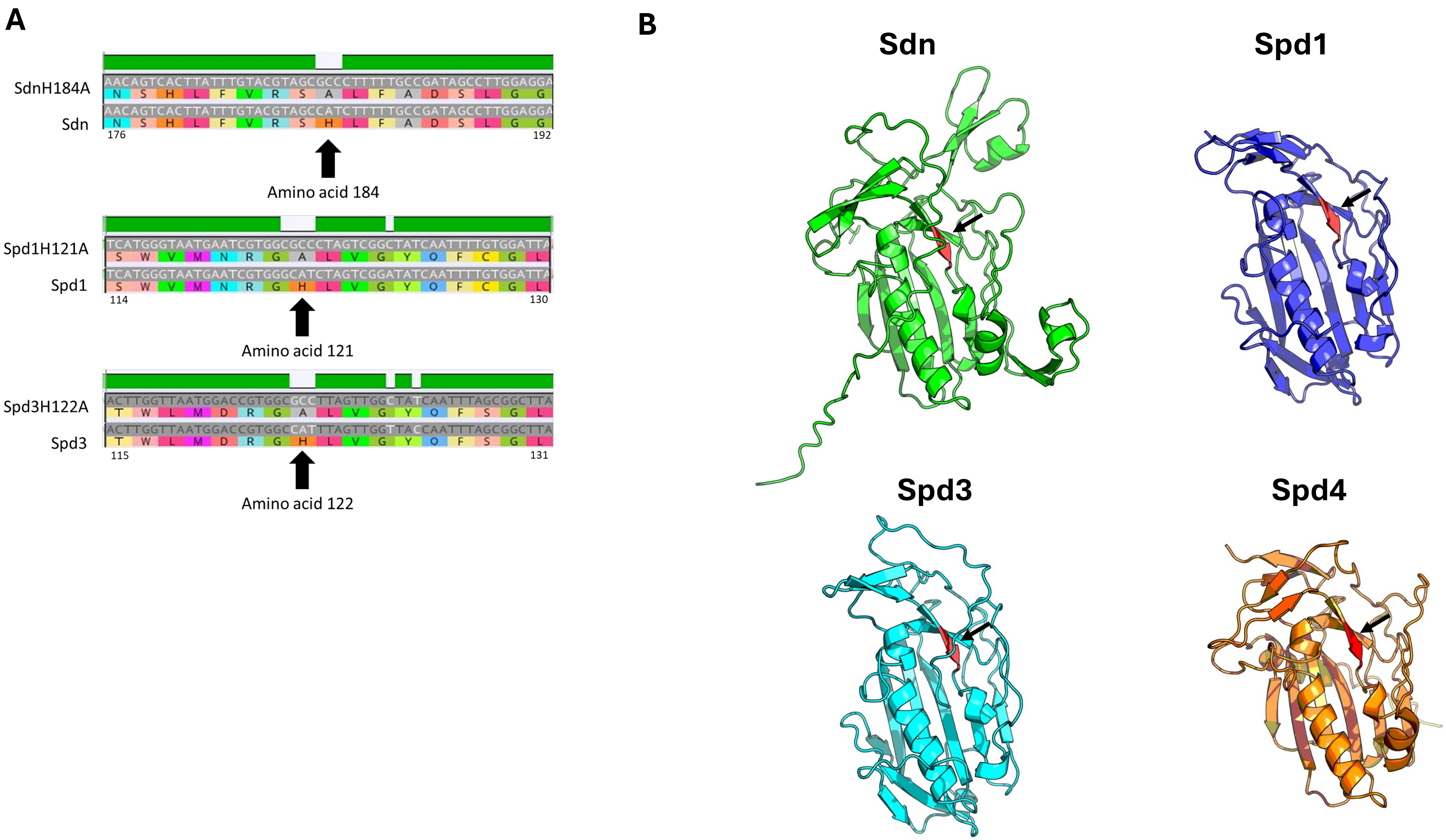

Fig.S4. A/ Alignment of WT and mutated version of Sdn, Spd1 and Spd3. The histidine residue in the catalytic site is indicated by the black arrow and was replaced by an alanine. **B/** AlphaFold2 [85,86] prediction of each LO1 DNase structure. The catalytic RGH-like motif is highlighted in red and with an arrow (the motif is buried in the Spd3 structure;

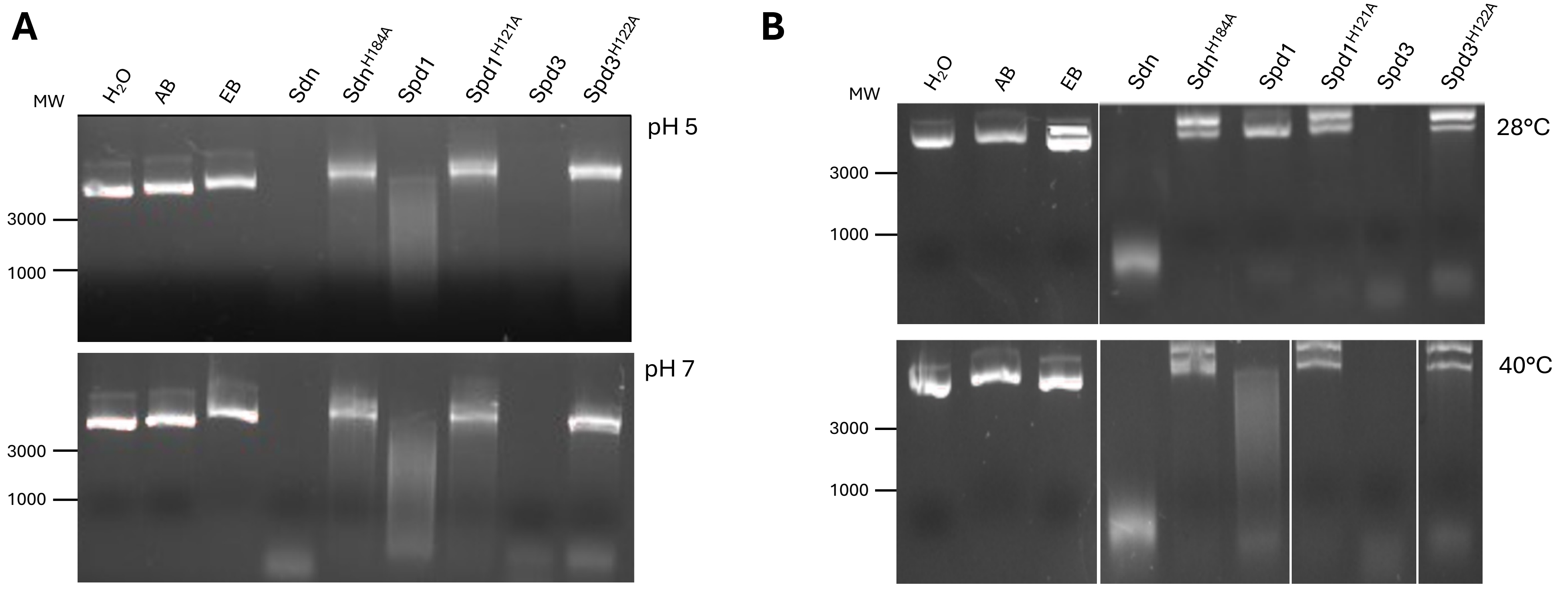

Fig.S5. Agarose gels electrophoresis of plasmid DNA incubated with the purified Spd1, Spd3 and Sdn as well as their mutated counterpart (Sdn^{H184A}, $Spd1^{H121A}$, $Spd3^{H122A}$) **(A)** at 37°C, pH 5 (upper) and pH 7 (lower panel) or (**B**) at 28°C (upper) and 40°C (lower panel) at pH 7. AB: activity buffer, EB: elution buffer. MW: molecular weight.

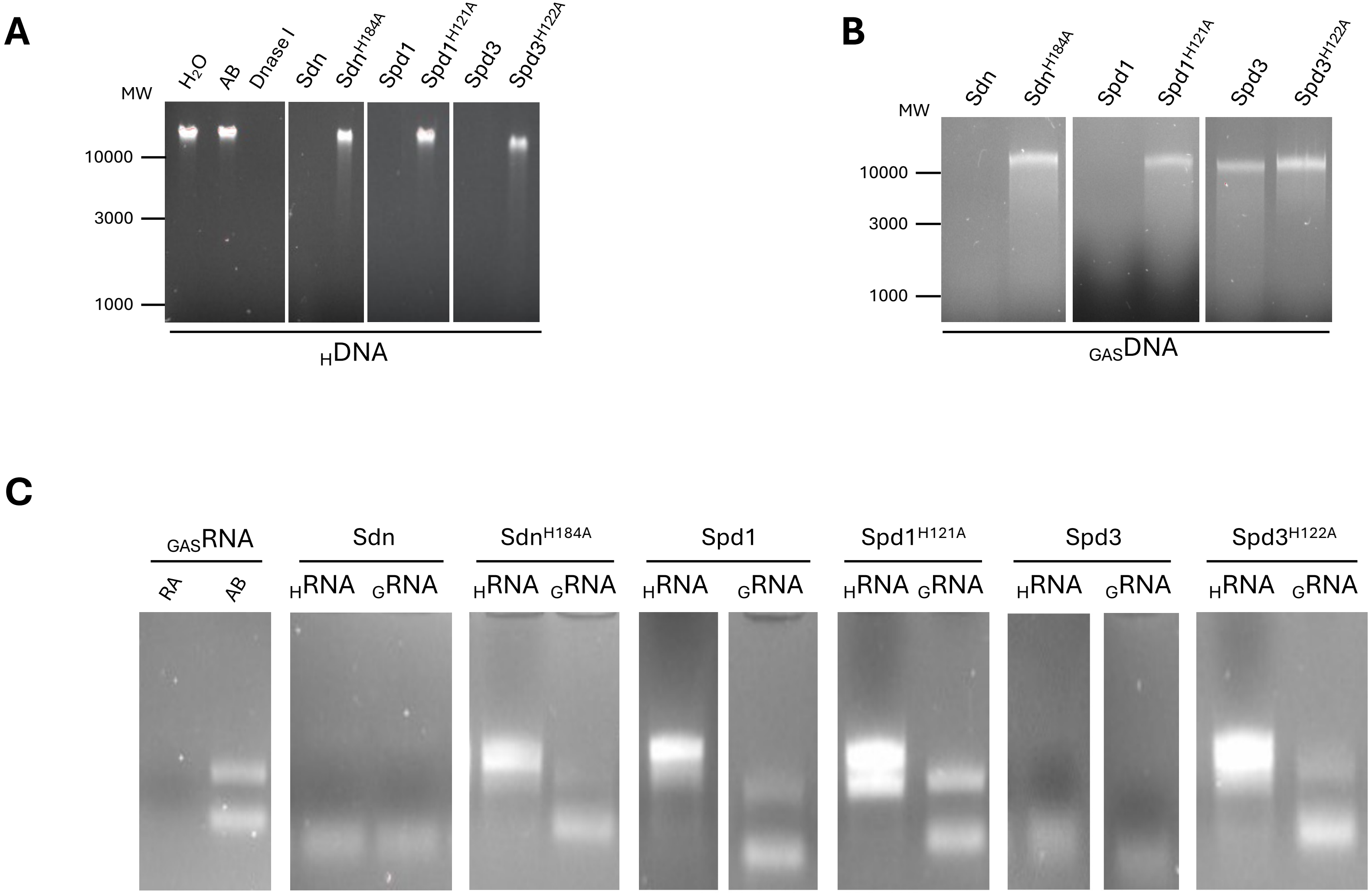

Fig.S6. Agarose gel electrophoresis of (**A**) genomic human DNA ($_{H}$DNA), (**B**) genomic GAS DNA ($_{GAS}$DNA) or (**C**) human ($_{H}$RNA) and GAS RNA ($_{GAS}$RNA) incubated with the purified Spd1, Spd3 and Sdn as well as their catalytic mutant (Sdn^{H184A}, Spd1^{H121A}, Spd3^{H122A}) at 37°C and pH 7. The DNaseI or RNase A (RA) have been used as positive controls. AB: activity buffer, MW: molecular weight.

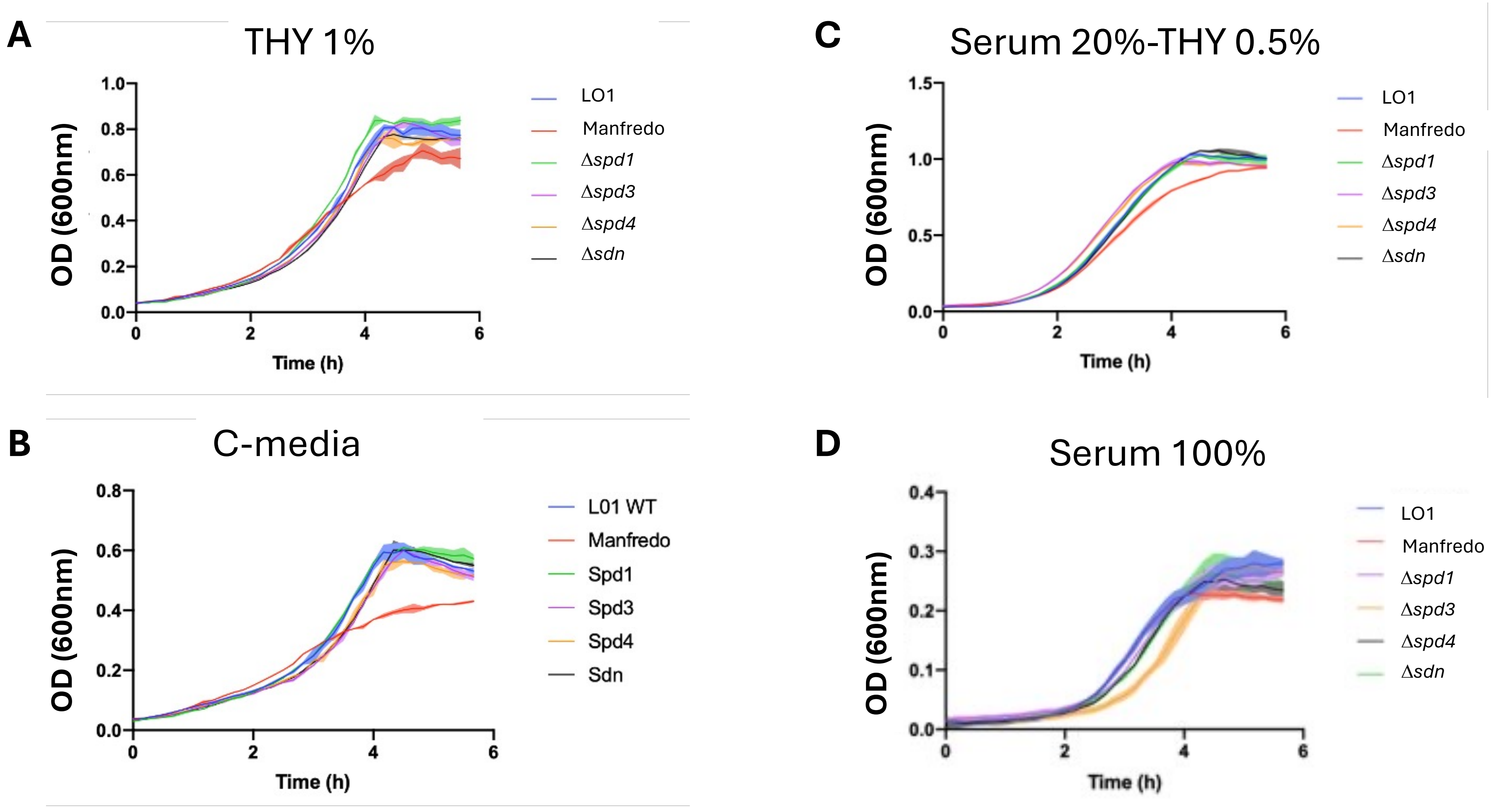

Fig.S7. Growth curves of the Manfredo strain, the LO1 and its mutants in THY (**A**), C-media (**B**), serum 20% (**C**) or 100% (**D**).

A

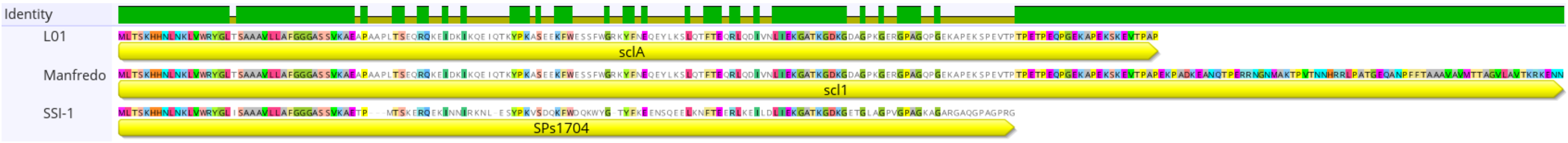

B

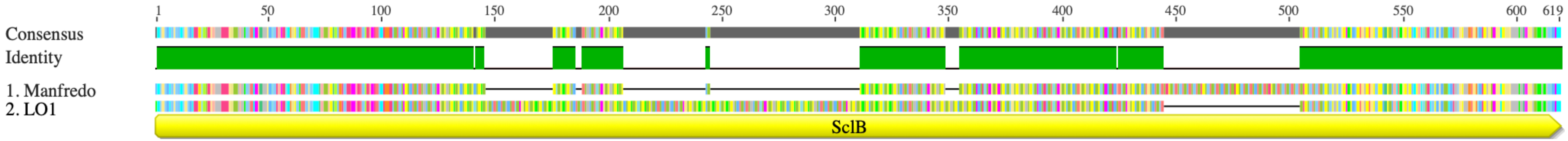

Fig.S8. Alignment of *sclA* (**A**) and *sclB* (**B**) from the Manfredo, LO1 and SSI-1 (M3 iGAS) strains using Geneious Prime® 2023.2.1.

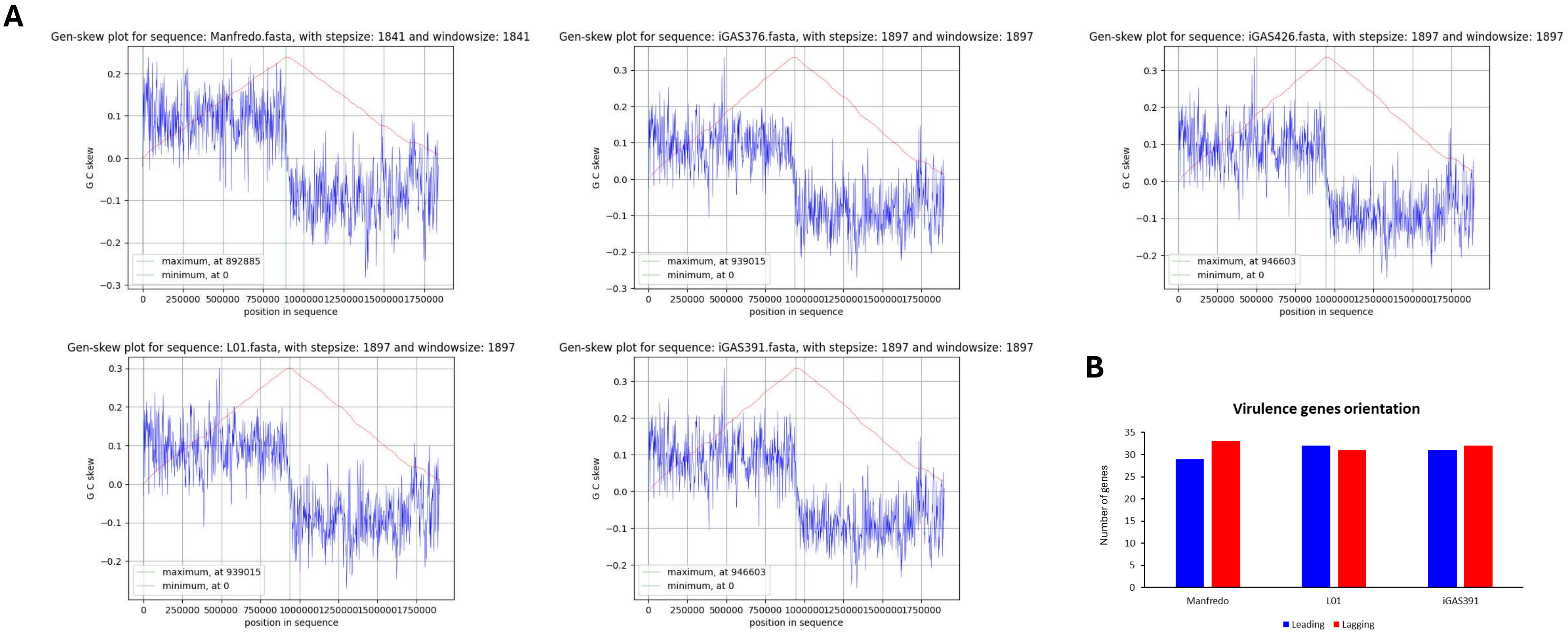

Fig.S9. (**A**) GC skew of the Manfredo, LO1 and the 3 Scottish iGAS genomes using GenSkew software (https://genskew.csb.univie.ac.at/) [87]. (**B**) Orientation of the virulence genes in the Manfredo, LO1 and iGAS391 strains.

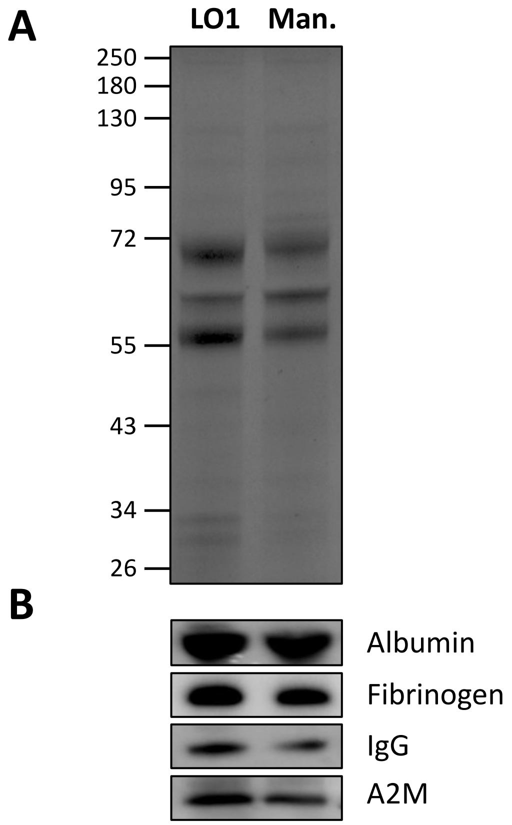

Fig.S10. Whole cell binding of the LO1 and Manfredo strains in human serum. (**A**) SDS-PAGE and Coomassie blue staining of eluted serum proteins from LO1 or Manfredo. (**B**) Immuno-detection of eluted serum proteins from LO1 or Manfredo with anti-albumin, -fibrinogen, -IgG and -alpha-2-macroglobulin antibodies.
