# Supplementary material for: Nosocomial Transmission of Necrotizing Fasciitis: A Molecular Characterization of Group A Streptococcal DNases in Clinical Virulence
Source: Microorganisms. 2024 Oct 31;12(11):2209. doi: 10.3390/microorganisms12112209 (PMC11596691; doi:10.3390/microorganisms12112209)
Supplement: Supplementary file 1 [file microorganisms-12-02209-s001.zip › Supplementary meth.pdf]

Supp data

RNA sequencing

Overnight cultures of GAS strains were grown to an OD_{600} of 0.3-0.4 (exponential phase, EP) or 0.8-1.0 (stationary phase SP). For serum and C medium, EP bacteria were centrifuged, washed with DPBS and resuspended in human serum or in C medium. Cultures were incubated for two additional hours. Then, 5 ml of cultures were centrifuged at 5000×g for 10 min. For biofilms formation, bacteria were grown for 3 days and biofilms were scrapped and centrifuged at 5000×g for 10 min. RNA protect buffer (NEB) was added to each pellet. Bacteria were lysed mechanically using matrix B beads with a FastPrep-24 5G™ (MP Biomedicals). For contact with human cells, cells were infected with EP bacteria in DMEM at a MOI of 100 for 2 hours at 37°C, 5% CO_2. Supernatant and scrapped cells were pooled and centrifuged at 5000×g for 10 min at 4°C. RNA protect buffer was added to the pellets and a differential lysis was performed. A first lysis with matrix D beads was performed to lyse human cells. Samples were centrifuged at 16000×g for 2 min and the supernatant was stored at -80°C for RNA extraction.

RNA Integrity Number (RIN) was determined using an Agilent Bioanalyzer 2100. rRNA were depleted from 200 ng of RNA using the NEBNext® rRNA Depletion Kit for bacteria (NEB). For samples incubated with human cells, human rRNA were further depleted with the NEBNext® rRNA Depletion Kit (Human/Mouse/Rat). Libraries were prepared with the NEBNext ultra II directional RNA library prep kit for Illumina (E7760L). The libraries were then sequenced with a NovaSeq 6000 system (Illumina).

RNA-seq analysis was performed with Geneious Prime® 2023.2.1 software. Paired-end reads were trimmed using BBDuk and mapped on the L01 (this work) or the Manfredo (NC_009332.1) genomes using Geneious mapper. The expression levels were calculated by counting reads as partial matches for multiple locations and compared using DESeq2.
